# Gut microbiota in genetic diseases: from Mendelian disorders to complex traits

**DOI:** 10.3389/fimmu.2026.1908644

**Published:** 2026-09-30

**Authors:** Guanjun Chen, Junrong Li, Tianjun Huang, Dan Zeng, Limin Mo, Jun Li, Chunjiang Zhu

**Affiliations:** The First Affiliated Hospital of Guilin Medical University, Guilin, China

**Keywords:** gut dysbiosis, gut microbiota, hereditary diseases, host-microbiota interaction, precision medicine

## Abstract

As a core component of the human microecosystem, the dynamic balance of the composition and function of gut microbiota is essential to host health. In recent years, numerous studies have demonstrated that gut dysbiosis is not only associated with metabolic diseases and immune disorders but also plays a pivotal role in the pathogenesis of hereditary diseases. Ranging from chromosomal diseases (e.g., Down syndrome), monogenic diseases (e.g., cystic fibrosis, phenylketonuria, familial adenomatous polyposis, hereditary hemolytic anemia), mitochondrial diseases (e.g., Leber’s hereditary optic neuropathy, mitochondrial encephalomyopathy) to polygenic hereditary diseases (e.g., inflammatory bowel disease, autism spectrum disorder, obesity and diabetes), gut microbiota can markedly alter the manifestation and severity of disease phenotypes by regulating host immune responses, metabolic pathways and gene expression. Nevertheless, the bidirectional interaction network between host genes and gut microbiota has not been fully elucidated. Most existing studies merely focus on correlation analysis, and in-depth exploration of causal mechanisms remains insufficient. This review elaborates on the mechanisms of gut microbiota in monogenic diseases and hereditary diseases with complex phenotypes. By integrating the latest advances in genomics, metabolomics and microbiomics, it discusses how gut dysbiosis affects disease progression via immune modulation, metabolite reprogramming and epigenetic modification. Meanwhile, this paper summarizes the current status and challenges of clinical translation of microbiota-targeted therapies, such as probiotics and fecal microbiota transplantation(FMT), in the treatment of hereditary diseases. This review aims to provide a microecological perspective for understanding the pathogenesis of hereditary diseases and offer theoretical support for personalized and precise intervention.

## 1 Introduction

Hereditary diseases are a category of diseases arising from alterations in genetic material, encompassing chromosomal diseases, monogenic diseases, mitochondrial diseases, and polygenic diseases with complex phenotypes. This review uniformly adopts the term “hereditary diseases” to emphasize their heritable nature and to encompass the full spectrum from Mendelian disorders to complex-trait diseases. These diseases are caused by gene mutations and exhibit a broad phenotypic spectrum, ranging from classic symptoms resulting from single-gene defects to complex phenotypes arising from the interplay of polygenic and environmental factors, manifesting substantial clinical heterogeneity. For instance, syndromic obesity is a rare complex disease associated with copy number variations, monogenic mutations and chromosomal abnormalities. Its phenotypes vary substantially among individuals, suggesting that environmental modifiers and epigenetic factors may play vital roles (1). Similarly, in early-onset inflammatory bowel disease (IBD), primary immunodeficiencies (PID) represent a group of monogenic diseases whose clinical manifestations are closely associated with genetic backgrounds. Mutations in the interleukin-10 receptor A (*IL-10RA*) and X-linked inhibitor of apoptosis protein (*XIAP*) are common causative factors in East Asian populations, and hematochezia is frequently the initial symptom in affected patients (2). Such complex mapping relationships between genotypes and phenotypes pose challenges to predicting disease progression merely based on genetic profiles.

In recent years, gut microbiota, as a critical mediator linking host genetics and environmental factors, has been proven to modulate the penetrance of gene mutations, disease severity and therapeutic responses. Gut dysbiosis has been found to be associated with a variety of hereditary diseases. In succinic semialdehyde dehydrogenase deficiency (SSADHD), mutations in the aldehyde dehydrogenase 5 family member A1 (*ALDH5A1*) gene trigger abnormal γ-aminobutyric acid (GABA) metabolism. Both patients and animal models present marked gut dysbiosis, including reduced alpha diversity and altered abundance of specific genera, indicating that gut microbiota may participate in the pathophysiology of this neurometabolic diseases (3). In autism spectrum disorder (ASD), gastrointestinal disturbances such as constipation and diarrhea are closely correlated with core ASD symptoms including irritability and social withdrawal. Gut dysbiosis contributes to the pathogenesis of ASD by regulating the gut-brain axis, immune responses and neurotransmitter metabolism (4, 5). Furthermore, in repeat expansion diseases (REDs) represented by Huntington’s disease, gut dysbiosis affects disease progression by modulating multiple biochemical pathways, which offers a novel perspective for exploring its complex etiology (6).

Gut dysbiosis not only affects neuropsychiatric hereditary diseases but also plays a pivotal role in other systemic diseases. For cutaneous diseases such as epidermolysis bullosa (EB), skin microbiome and metagenomic analyses have opened new avenues for precision medicine and uncovered the interactions between genetic mutations and microbial composition (7). In cardiovascular diseases, gut dysbiosis promotes the development of atherosclerosis, myocardial infarction and other diseases by producing metabolites such as trimethylamine N-oxide (TMAO) and lipopolysaccharide (LPS) (8). Similarly, reduced diversity and structural disturbance of gut microbiota are closely associated with elevated blood pressure in hypertension, and FMT experiments have verified the causal regulatory effect of microbiota on blood pressure (9).

In-depth exploration of the roles of gut microbiota in hereditary diseases not only helps elucidate the full pathway linking genotypes to phenotypes but also lays a scientific foundation for the development of microbiota-based intervention strategies. For example, allogeneic hematopoietic stem cell transplantation can reverse gut dysbiosis and relieve symptoms in patients with early-onset IBD (2). In the context of ASD, gut microbiota-targeted interventions, including probiotics and FMT, have shown potential in alleviating behavioral manifestations (5). Modulation of gut microbiota has emerged as a promising therapeutic approach for obesity and metabolic diseases (10, 11). In addition, gut microbiota can modulate therapeutic responses by interfering with drug metabolism, which involves the joint metabolism of drugs by hepatic drug-metabolizing enzymes and intestinal microbes (12). Accordingly, systematically elaborating the roles of gut microbiota in hereditary diseases, with a focus on its molecular mechanisms, clinical correlations and therapeutic potential, is of great significance for advancing precision medicine and personalized medicine. **Table 1** summarizes the bidirectional regulatory effects of the gut microbiota across different categories of hereditary diseases from a disease-classification perspective, providing an overarching framework for the detailed discussions in subsequent sections.

**Table 1:** Comparison of bidirectional regulatory effects of the gut microbiota across different categories of hereditary diseases.

Representative disease	Host genetic defect	Genetic shaping of microbiota	Microbiota-mediated phenotypic modification	Disease-specific mechanism
Down syndrome	Trisomy 21 (gene dosage effect) (13)	*Firmicutes*/*Bacteroidete*s ↓, *Proteobacteria* ↑; beneficial bacteria ↓, pathogenic bacteria ↑ (14–16)	Severity of cognitive impairment, rate of premature aging, susceptibility to infection (17–20)	SFB-induced Th17 differentiation (17)
Cystic fibrosis	CFTR mutation (21)	*Proteobacteria* ↑; selective proliferation of *Pseudomonas aeruginosa (*22)	Rate of lung function decline, degree of nutrient malabsorption (23–27)	Mucus hyperviscosity → altered anaerobic microenvironment; gut–lung axis bile acid signaling (28, 29)
Familial adenomatous polyposis	APC germline mutation (30)	*Firmicutes*/*Bacteroidetes* ↑, *Proteobacteria* ↑ (31, 32)	Polyp count, age of malignant transformation, tumor burden (30, 31)	ETBF toxin, colibactin-induced mutagenesis; secondary bile acid–mediated ROS (30, 33–37)
Phenylketonuria	PAH mutation (38)	Butyrate-producing bacteria ↓, microbiota diversity ↓; selective pressure from low-Phe diet (39, 40)	Blood Phe level fluctuation, degree of neurological damage (38, 41)	Microbiota Phe metabolic bypass (deaminase) (41–43)
MELAS/MERRF/LHON	mtDNA mutation (e.g.,m.3243A>G) (44, 45)	Butyrate-producing bacteria ↓, pro-inflammatory bacteria ↑ (46)	Severity of neuromuscular symptoms, mutation load, degree of optic nerve atrophy (47, 48)	Respiratory chain complex I/IV inhibition; peroxynitrite-mediated damage (47, 49)
Inflammatory bowel disease	Polygenic risk including NOD2, etc. (50, 51)	α-diversity ↓, Firmicutes ↓, *Proteobacteria*↑ *(*52–54)	Disease activity, drug response, postoperative recurrence, cancer risk (55, 56)	AIEC invasion, biofilm formation, CEACAMs-mediated adhesion (57–60)
Autism spectrum disorder	Polygenic risk + *de novo* mutations (61)	*Clostridium* ↑, *Faecalibacterium prausnitzii* ↓, *Akkermansia* ↓ (62–64)	Severity of social impairment, frequency of GI symptoms, IQ level (61–63, 65, 66)	Intestinal hyperpermeability (leaky gut), claudin-5 downregulation, blood–brain barrier disruption (67–71)
Obesity/Type 2 diabetes	Polygenic susceptibility (72)	F/B ratio ↑, butyrate-producing bacteria ↓, LPS/TMAO-producing bacteria ↑ (73–77)	Rate of weight gain, degree of insulin resistance, complication risk (76–79)	FXR bile acid signaling dysregulation (80)

## 2 Fundamentals of the interaction between gut microbiota and host genetics

### 2.1 Regulation of gut microbiota composition by host genes

Host genes shape gut microbiota colonization, diversity, and homeostasis by encoding immune-related molecules and intestinal barrier proteins. For example, Toll-like receptor 4 (TLR4), a key pattern recognition receptor, influences intestinal barrier integrity and microbial immune tolerance; TLR4 mutation alleviates gut dysbiosis and renal inflammation in hypertensive models (81, 82). Similarly, monogenic immune defects such as interleukin-10 (IL-10) receptor deficiency disrupt host–commensal balance, leading to mucosal permeability and intestinal inflammation (83).

Genome-wide association studies (GWAS) and microbiome-wide association studies (mGWAS) have identified host loci that influence microbial composition. In humans, loss-of-function mutations in fucosyltransferase 2 (FUT2)—the “non-secretor” phenotype linked to IBD risk—alter the abundance of mucosa-associated bacteria (84–86). Cross-ethnic mGWAS (HUNT, Dutch Microbiome Project, Chinese cohorts) further confirmed that functional loci such as lactase (LCT) and ABO blood group (ABO) exert directional selection on microbial metabolic functions (87–89). These findings demonstrate that host genotype, from monogenic defects to polygenic backgrounds, is a core determinant of gut microbiota composition and function.

### 2.2 Epigenetic regulation of host gene expression by gut microbiota

Gut microbiota exerts epigenetic regulation on host gene expression through metabolites such as short-chain fatty acids(SCFAs) and bile acids (90, 91). Butyrate, a major SCFA, acts as a histone deacetylase (HDAC) inhibitor that remodels chromatin into a transcriptionally permissive state, thereby upregulating forkhead box P3 (FOXP3) and driving regulatory T cells (Treg) differentiation to maintain immune tolerance (92). Microbial metabolites also modulate DNA methylation; dysbiosis-induced aberrant epigenetic modifications—including histone acetylation, DNA methylation, and non-coding RNA-mediated gene silencing—may directly aggravate the severity of hereditary diseases (93, 94).

Such epigenetic alterations can silence protective genes and accelerate disease progression (95). In IBD, for example, dysbiosis-induced changes in microbial metabolites can trigger DNA methylation or histone modifications in the promoter regions of intestinal barrier genes (e.g., tight junction proteins), leading to barrier disruption, translocation of antigens and pathogens, and excessive inflammation, particularly in genetically susceptible individuals (96–99). Microbial metabolites such as indolepropionic acid and lithocholic acid also regulate cytochrome P450 3 (ACYP3A) transcription via pregnane X receptor (PXR), with further modulation by indoxyl sulfate, illustrating the complexity of microbiota-mediated host regulation (100).

Gut microbiota also regulates host gene expression through non-coding RNAs and RNA N^6^-methyladenosine (m6A) methylation, affecting post-transcriptional control and inflammatory responses (101–104). Collectively, these multi-level epigenetic mechanisms—histone modification, DNA methylation, non-coding RNA regulation, and RNA methylation—form a dynamic bridge linking environmental factors to host gene expression; their dysregulation contributes to phenotypic diversity and disease severity in complex hereditary diseases.

### 2.3 Gut microbiota as a bridge between genetic and environmental factors

Gut microbiota acts as a dynamic mediator between host genetics and environment. Environmental exposures—such as diet, antibiotics, and altitude—reshape microbial composition, which in turn amplifies or mitigates genetic risks and modulates complex phenotypes (105). In polygenic diseases such as obesity and type 2 diabetes, high-fat diet-induced gut dysbiosis exacerbates metabolic diseases and autism-like behaviors by altering tryptophan metabolism and serotonin synthesis (106). FMT confirms this causal link: germ-free mice receiving microbiota from obese donors rapidly develop weight gain and insulin resistance, demonstrating that microbial composition can transfer metabolic traits (107).

As a gene–environment bridge, gut microbiota also offers intervention targets. Probiotics or FMT may partially reverse pathological phenotypes caused by genetic defects or adverse exposures, supporting precision medicine approaches (108, 109).

### 2.4 Cross-disease shared microbiota–host interaction pathway framework

#### 2.4.1 *TLR4/NF-κB* inflammatory cascade pathway

Gut dysbiosis, particularly the overgrowth of Gram-negative bacteria leads to increased release of LPS. As a pathogen-associated molecular pattern, LPS is recognized by TLR4 on the surface of immune cells, thereby activating the nuclear factor-κB (NF-κB) signaling pathway and promoting the production of pro-inflammatory cytokines such as tumor necrosis factor-α (TNF-α) and interleukin-6 (IL-6) (110, 111). This pathway plays a central role across multiple systemic diseases, including metabolic, neurological, and immune diseases (**Figure 1A**).

**Figure 1:**
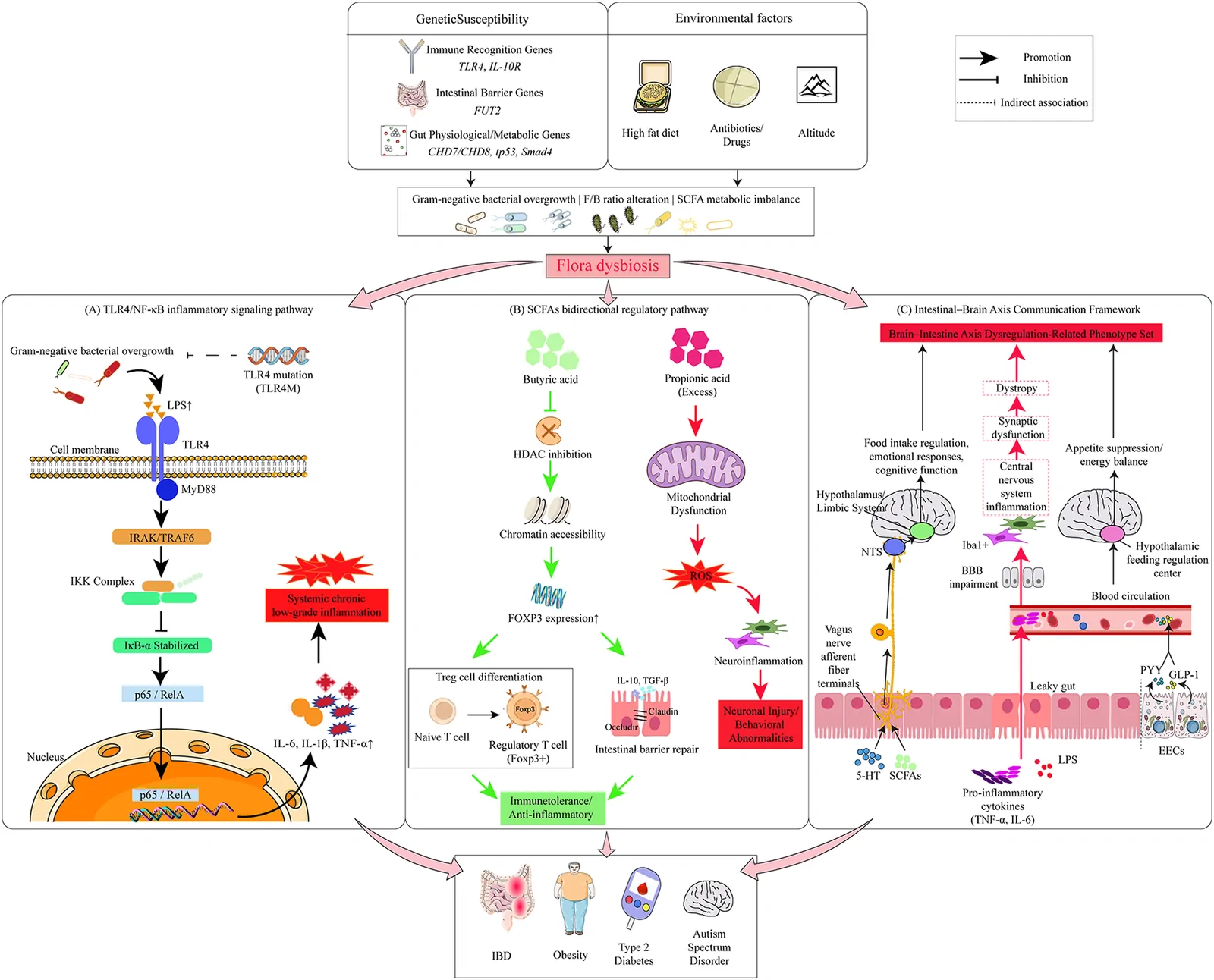
Shared microbiota-host interaction network across diverse diseases. Host genetic factors (e.g., monogenic defects in TLR4, IL-10R, FUT2, CHD7/8, TP53) and environmental factors (diet, antibiotics, high-fat diet) jointly shape gut microbiota composition. Impaired immune surveillance and intestinal barrier integrity trigger gut dysbiosis, featured by expansion of Gram-negative bacteria (e.g., phylum *Proteobacteria*), altered *Firmicutes*/*Bacteroidetes* ratio, and disrupted SCFA metabolism. Dysbiotic microbiota serves as the common upstream trigger for three major signaling cascades. **(A)** TLR4/NF-κB inflammatory cascade: Gut dysbiosis elevates LPS release. LPS is recognized by TLR4 on immune-cell surfaces and initiates intracellular MyD88/IRAK/TRAF6 signaling, leading to IKK-complex activation, IκBα degradation and NF-κB nuclear translocation. Nuclear NF-κB drives transcription of pro-inflammatory cytokines including TNF-α and IL-6, resulting in systemic chronic low-grade inflammation. TLR4 mutant (TLR4M) confers protective effects against dysbiosis-mediated injury. **(B)** Bidirectional regulation by SCFAs: In the protective branch (green), butyrate inhibits HDAC, promotes chromatin remodeling, up-regulates FOXP3 expression, drives Treg differentiation and enhances intestinal-barrier repair, thereby mediating immune tolerance and anti-inflammatory responses. In the pathogenic branch (red), excessive propionic acid induces mitochondrial dysfunction and ROS production, triggering neuroinflammation followed by neuronal damage or behavioral abnormalities. **(C)** Gut-brain-axis communication: Three parallel signaling routes connect the gut and brain. Neural pathway: microbial metabolites such as SCFAs and 5-HT are sensed by vagal afferent fibers, relayed via the nucleus tractus solitarius (NTS), and projected to the hypothalamus and limbic system to regulate feeding, mood and cognition. Endocrine pathway: gut EECs secrete GLP-1 and PYY in response to microbial cues; these hormones travel via systemic circulation to the hypothalamic feeding center to control appetite and energy homeostasis. Immune-inflammatory pathway: LPS and pro-inflammatory cytokines translocate across the compromised intestinal barrier into peripheral circulation, penetrate the disrupted BBB, activate microglia and astrocytes, and induce central neuroinflammation as well as synaptic dysfunction. Outputs of the three pathways converge on three major disease categories: metabolic diseases (obesity, type 2 diabetes mellitus), neuropsychiatric disorders (ASD), and immune-inflammatory diseases (IBD). This schematic incorporates the following third-party icons, reproduced without modification: the "brain" icon by Servier (https://smart.servier.com/ is licensed under CC-BY 3.0 Unported https://creativecommons.org/licenses/by/3.0/); and "gene" icon by DBCLS (https://togotv.dbcls.jp/en/pics.html is licensed under CC-BY 4.0 Unported https://creativecommons.org/licenses/by/4.0/).

#### 2.4.2 Bidirectional regulatory effects of SCFAs

SCFAs are the principal metabolites produced by microbiota-mediated fermentation of dietary fiber; among these, butyrate, propionate, and acetate serve as the core effector metabolites capable of entering immune organs such as the bone marrow and spleen, thereby influencing immune cell differentiation and function (112). Butyrate exerts anti-inflammatory and intestinal barrier–protective effects through inhibition of histone deacetylases (HDACs). Conversely, excessive propionate can induce neuroinflammation and disrupt mitochondrial function, demonstrating a dose- and context-dependent dual effect (113, 114) (**Figure 1B**).

#### 2.4.3 Gut–brain axis communication framework

The gut–brain axis constitutes a bidirectional communication network linking the intestine and the central nervous system, primarily relying on three parallel pathways for signal transduction. First, the neural pathway mediates rapid signal transmission: afferent vagal fibers can directly recognize microbiota-derived metabolic signals such as SCFAs and 5-hydroxytryptamine (5-HT) relaying these signals sequentially to the nucleus tractus solitarius before ultimately projecting to key brain regions including the hypothalamus and limbic system (115). Beyond immediate neural transmission, the endocrine pathway is responsible for long-lasting humoral signal delivery: enteroendocrine cells (EECs), upon sensing microbiota alterations, release peptide hormones such as glucagon-like peptide-1 (GLP-1) and peptide YY (PYY), which reach the hypothalamic feeding-regulatory center via the bloodstream to exert their effects (116). Distinct from the modulatory patterns mediated by neural and hormonal signaling, the immune-inflammatory pathway predominantly transmits pathological signals: microbiota-derived products such as LPS and various cytokines translocate across a compromised intestinal barrier into the peripheral circulation, eliciting a systemic inflammatory response, whereby abundant pro-inflammatory factors breach the blood–brain barrier and subsequently induce central neuroinflammation (117, 118) (**Figure 1C**).

## 3 Characteristics of gut dysbiosis in patients with chromosomal disorders

Chromosomal diseases arise from numerical or structural chromosomal abnormalities, manifesting as multisystem dysfunction including intellectual disability, impaired immunity, and metabolic or gastrointestinal disturbances (13, 119). Among these, Down syndrome (DS)—caused by trisomy 21, the most common numerical chromosomal aberration—is the most prevalent form (13).

### 3.1 Alterations in gut microbiota composition in patients with Down syndrome

Patients with DS exhibit prominent structural imbalance in the composition of gut microbiota (**Figure 2**). Studies have demonstrated that, compared with healthy controls, individuals with DS exhibit reduced overall gut microbiota diversity, with a significantly decreased *Firmicutes*/*Bacteroidetes* ratio and elevated *Proteobacteria* abundance at the phylum level; metagenomic analysis further reveals concomitant downregulation of gene abundance related to carbohydrate metabolism and SCFA biosynthesis, which collectively compromise host intestinal barrier integrity and energy metabolic homeostasis (14). These changes are closely linked to recurrent infections and gastrointestinal symptoms commonly observed in DS patients (15). For example, a study on Chinese children with DS found that the species composition of *Blautia* is correlated with cognitive function. The abundances of *Blautia massiliensis* and *Blautia argi* are decreased, whereas *Blautia faecis* is enriched. In addition, acetate, a metabolite of *Blautia*, is significantly reduced in the DS group (16). These findings demonstrate that gut dysbiosis in DS patients not only alters the abundance of specific microbes, but also profoundly affects microbial metabolic functions, which may aggravate core symptoms such as cognitive impairment.

**Figure 2:**
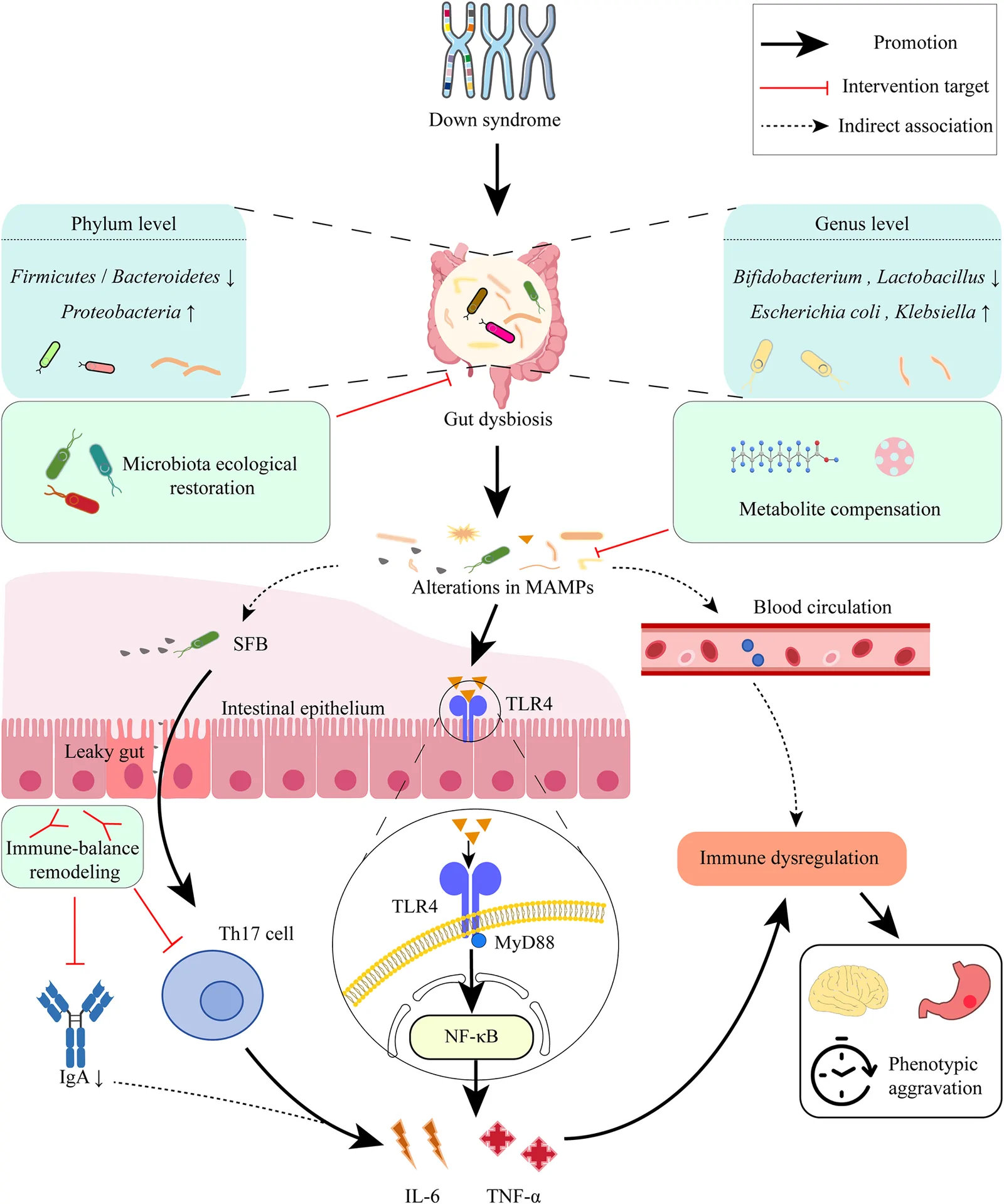
Schematic illustration of how gut dysbiosis in DS drives cognitive and related phenotypes via immunological pathways. DS induces characteristic gut microbiota dysbiosis. At the phylum level, it is featured by a decreased *Firmicutes*/*Bacteroidetes* ratio and elevated abundance of *Proteobacteria*. At the genus level, beneficial taxa including *Bifidobacterium* and *Lactobacillus* are depleted, whereas opportunistic pathogens such as *Escherichia coli* and *Klebsiella* are enriched. Gut dysbiosis alters MAMPs and triggers leaky-gut phenotype. MAMPs activate the epithelial TLR4/NF-κB signaling cascade across the impaired intestinal barrier, driving the secretion of pro-inflammatory cytokines IL-6 and TNF-α. Local intestinal immunity is disrupted: reduced secretory IgA and expanded SFB promote Th17-cell differentiation, further amplifying local intestinal inflammation. MAMPs and pro-inflammatory mediators enter the bloodstream and cause systemic immune imbalance, ultimately aggravating multisystem clinical phenotypes of Down syndrome. Three categories of intervention targets are illustrated in this image: microbiota ecological restoration, metabolite compensation, and immune-balance remodeling. They block pathological cascades at the levels of microbial community structure, metabolites and immune responses respectively, and provide potential therapeutic strategies for Down-syndrome-related complications. This schematic incorporates the following third-party icons, reproduced without modification: the "chromosome-1" icon by Servier (https://smart.servier.com/ is licensed under CC-BY 3.0 Unported https://creativecommons.org/licenses/by/3.0/).

### 3.2 Gut microbiota modulates the phenotypes of chromosomal disorders via immune mechanisms

The intestinal mucosal immune system serves as the first line of host defense against pathogenic microorganisms, and its functional homeostasis is closely associated with the composition of gut microbiota (**Figure 2**). In patients with chromosomal diseases, intestinal mucosal barrier function is frequently compromised, and dysbiosis-induced alterations in microbe-associated molecular patterns (MAMPs) subsequently trigger local inflammatory responses (8). For example, reduced intestinal immunoglobulin A (IgA) secretion and elevated proportion of T helper 17 (Th17) cells are common immunological abnormalities in patients with DS. Studies have shown that the abundance of *segmented filamentous bacteria* (SFB) is markedly increased in the gut of DS patients. As a well-known potent inducer of Th17 cells, elevated SFB abundance is closely correlated with aggravated local pro-inflammatory status in the intestine (17). Accordingly, gut dysbiosis in patients with chromosomal diseases exacerbates local intestinal and systemic immune disorders by impairing mucosal barriers, activating innate immunity and regulating the differentiation of adaptive immune cells.

The gut microbiota not only influences local immunity, but its metabolites can also remotely modulate systemic immune homeostasis via the bloodstream. This remote regulatory mechanism may become dysregulated in patients with chromosomal diseases. For example, individuals with DS frequently exhibit gut dysbiosis, with human cohort studies confirming that the abundance of pro-inflammatory taxa such as *Escherichia/Shigella* is significantly elevated and positively correlates with systemic inflammatory levels (17). Such alterations in microbial composition generally lead to elevated levels of pro-inflammatory cytokines in peripheral blood, including IL-6 and TNF-α (18–20). Chronic systemic low-grade inflammation is recognized as a critical driver of cognitive decline and premature aging phenotypes in DS patients, and gut dysbiosis plays an essential part in this process (120). Therefore, the remote regulation of the systemic immune system by gut microbiota-derived metabolites may serve as a vital bridge linking intestinal microecology to systemic phenotypes of chromosomal diseases.

## 4 The role of gut microbiota in monogenic disorders

### 4.1 Cystic fibrosis

#### 4.1.1 Remodeling of the intestinal microenvironment by *CFTR* mutations

CF is an autosomal recessive genetic disease caused by mutations in the cystic fibrosis transmembrane conductance regulator (*CFTR*) gene. The core pathogenesis lies in the dysfunction of CFTR protein, which impairs the transport of chloride and bicarbonate ions and consequently leads to viscous secretions in multiple organs throughout the body (21). In the intestine, such abnormal ion transport directly disrupts the normal hydration of the mucus layer and creates a highly viscous intestinal microenvironment. This unique microenvironment provides niches for the overgrowth of specific bacteria, particularly opportunistic pathogens belonging to Proteobacteria, such as *Pseudomonas aeruginosa* (22). Studies have demonstrated that *CFTR* gene mutations alone can drive the selective alteration of gut microbiome. Even in germ-free mouse models, *CFTR* deficiency reshapes the composition of gut microbiota independent of exogenous infections, confirming that genetic defects are one of the fundamental causes of gut dysbiosis (22).

#### 4.1.2 Modification and heterogeneity of multi-organ phenotypes in CF by gut dysbiosis

Gut dysbiosis is not an isolated event; it forms a mutually reinforcing vicious cycle with local inflammatory responses. In CF, dysbiotic microbiota, particularly the overgrowth of members within phylum *Proteobacteria*, release cell-wall components such as LPS, which exacerbate intestinal inflammation via the TLR4/NF-κB signaling cascade (23). Meanwhile, persistent inflammation further impairs the integrity of the intestinal epithelial barrier and disrupts the absorption of fat-soluble vitamins (vitamin A, D, E and K) by intestinal cells, resulting in malnutrition and impaired systemic immune regulation (24). This cycle of “dysbiosis-inflammation-malabsorption” represents the core feature of intestinal pathophysiology in CF (**Figure 3A**).

**Figure 3:**
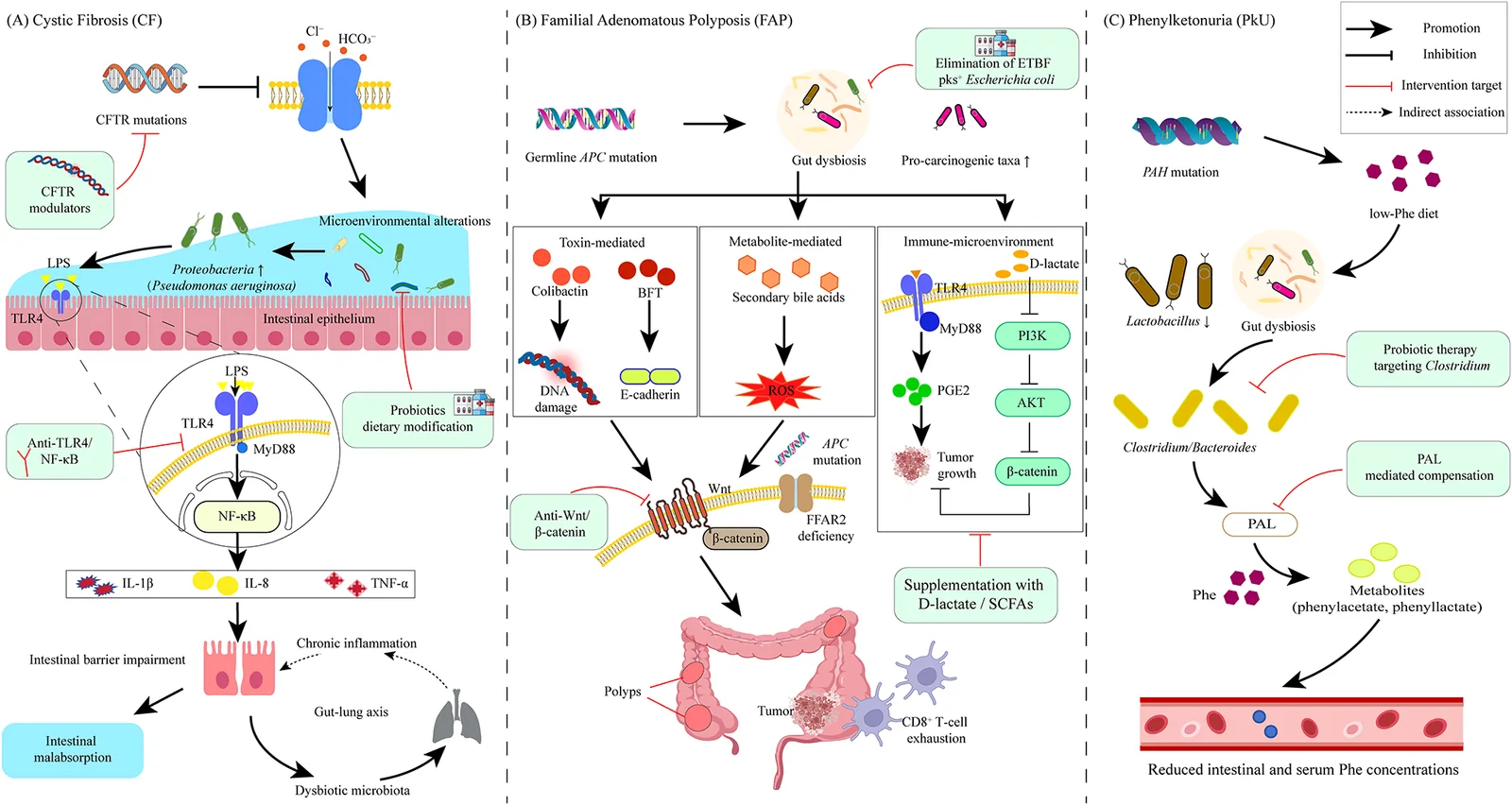
Multiple pathogenic mechanisms of gut microbiota in monogenic disorders. **(A)** CF: *CFTR* gene mutations suppress chloride and bicarbonate transport, alter the intestinal microenvironment, and drive the enrichment of *Pseudomonas aeruginosa*. Microbiota-derived LPS activates the epithelial TLR4-NF-κB signaling pathway and induces the secretion of pro-inflammatory cytokines including IL-1β, IL-8 and TNF-α, triggering intestinal barrier injury and intestinal malabsorption. A vicious cycle of chronic inflammation and dysbiosis is formed via the gut-lung axis. Potential intervention targets include CFTR modulators, TLR4/NF-κB pathway inhibitors, probiotics and dietary adjustment. **(B)** FAP: Germline *APC* mutations provoke gut microbiota dysbiosis with increased abundance of pro-carcinogenic taxa, which promote intestinal polyp and tumor development through three pathways. ① Toxin-mediated pathway: BFT and colibactin damage epithelial E-cadherin and induce DNA damage respectively, leading to activation of the Wnt/β-catenin pathway. ② Metabolite-mediated pathway: Secondary bile acids trigger ROS production and cooperatively activate pro-oncogenic cascades. ③ Immune microenvironment pathway: D-lactate activates the PI3K/AKT/β-catenin axis to suppress tumor proliferation, while gut microbiota can trigger the TLR4/MYD88 signaling cascade to promote PGE2 release, thereby accelerating tumor growth. FFAR2 deficiency induces CD8^+^ T-cell exhaustion. Feasible intervention strategies include eliminating ETBF-producing Bacteroides fragilis and colibactin-producing pathogenic Escherichia coli, inhibiting the Wnt/β-catenin pathway, and supplementing D-lactate as well asSCFAs. **(C)** PKU: Mutations in the *PAH* gene impair phenylalanine metabolism, and a low-phenylalanine diet can modulate disease progression. Such mutations are accompanied by depletion of *Lactobacillus* and gut microbial disturbance. A *Clostridium*/*Bacteroides*-mediated metabolic shunt converts phenylalanine into phenylacetate, phenyllactate and other metabolites through the PAL-dependent compensatory pathway, lowering phenylalanine concentrations in the gut and peripheral blood. Intervention approaches include Clostridium-targeted probiotic therapy and PAL-based compensatory therapy. This schematic incorporates the following third-party icons, reproduced without modification: the "damaged"_DNA icon by Rahel-Khdhr (https://linktr.ee/rahe1 is licensed under CC-BY 4.0 Unported https://creativecommons.org/licenses/by/4.0/) and the "A-DNA" icon by DBCLS (https://togotv.dbcls.jp/en/pics.html is licensed under CC-BY 4.0 Unported https://creativecommons.org/licenses/by/4.0/).

Clinical studies have further revealed a close association between gut microbiota and multi-organ phenotypes in CF. Multiple studies have verified that the alpha diversity of gut microbiota in CF patients is positively correlated with pulmonary function indicators such as forced expiratory volume in one second as a percentage of predicted value (FEV1%), and negatively correlated with airway inflammatory markers including interleukin-8 (IL-8) and neutrophil elastase (25, 26). For instance, a longitudinal study on children with CF demonstrated that those dominated by *Bifidobacterium* in the gut had a markedly lower incidence of pulmonary exacerbations compared with children with predominant *Bacteroides*. Meanwhile, the former group presented better pulmonary function and milder intestinal inflammation (27). In addition, bile acids, as signaling molecules co-metabolized by the host and microbiota, have been found to be closely associated with airway inflammation and alterations in microbial community structure in pulmonary secretions of CF patients. This further confirms the pivotal role of the gut-lung axis in CF disease progression (28, 29). These findings indicate that gut microbiota-targeted interventions, such as supplementation with probiotics including *Lactobacillus rhamnosus* GG strain or dietary modification, may reshape intestinal microecology and alleviate inflammation, thereby exerting beneficial effects on clinical outcomes of the lungs and other organs in CF patients (121).

### 4.2 Carcinogenic mechanisms of gut microbiota in familial adenomatous polyposis

#### 4.2.1 *APC* mutation–driven selection of pro-carcinogenic microbiota

FAP is an autosomal dominant hereditary disease caused by germline mutations in the adenomatous polyposis coli (*APC*) gene. Nearly all patients develop colorectal cancer (CRC) before the age of 40 (30). *APC* mutations not only drive aberrant intestinal epithelial proliferation through the Wnt/β-catenin pathway but also reshape gut microbiota ecology. In FAP patients, gut microbiota composition undergoes marked alterations, characterized by an elevated *Firmicutes/Bacteroidetes* ratio and increased *Proteobacteria* abundance; such dysbiosis is closely associated with polyp burden (31). Whole-exome sequencing combined with metagenomic analysis of 35 patients with intestinal adenomas revealed that individuals harboring highly pathogenic *APC* mutations exhibited significant enrichment of *Fusobacterium mortiferum* and marked depletion of butyrate-producing *Faecalibacterium prausnitzii* and *Bifidobacterium pseudocatenulatum;* moreover, the *APC* mutation group showed significantly reduced abundance of microbial photosynthesis pathways, suggesting that host genetic defects directly drive reprogramming of microbiota energy metabolism (32). Notably, even among FAP patients carrying identical *APC* mutations (e.g., exon 15 mutations), gut microbiota composition remains significantly heterogeneous, indicating that the microbiota serves as a critical environmental modifier of polyp burden and malignant transformation risk.

#### 4.2.2 Modification of FAP phenotypic progression by microbiota-mediated carcinogenic mechanisms

Recent studies have revealed that gut microbiota plays a critical role in *APC* mutation-driven tumorigenesis, and its carcinogenic mechanisms involve the combined effects of specific bacterial toxins, metabolites and immune microenvironment. For example, enterotoxigenic Bacteroides fragilis (ETBF)–secreted Bacteroides fragilis toxin (BFT) and *Escherichia coli (E. coli*)–derived colibactin can respectively accelerate polyp formation by cleaving E-cadherin to activate the Wnt/β-catenin pathway and exacerbate genomic instability by inducing DNA double-strand breaks, thereby cooperatively driving the malignant transformation of adenomas in FAP (30). Microbial metabolites also exert vital effects during carcinogenesis in FAP. As metabolites of cholesterol degraded by gut microbiota, secondary bile acids induce reactive oxygen species (ROS) production and DNA oxidative damage, and cooperate with *APC* mutations to activate the Wnt/β-catenin pathway (33). Studies have demonstrated that in *ApcMin/+* mouse models, gut microbiota activates the TLR4/MYD88 (myeloid differentiation primary response 88) signaling pathway, promotes the metabolism of arachidonic acid into prostaglandin E2 (PGE2), and consequently accelerates tumor growth (34). Notably, deficiency of the SCFA receptor free fatty acid receptor 2 (FFAR2) impairs intestinal barrier integrity, increases intratumoral bacterial load and induces CD8+ T cell exhaustion, eventually facilitating colon tumorigenesis (35). In addition, D-lactic acid, a microbial metabolite, exerts chemopreventive effects in *ApcMin/+* mice via inhibiting the phosphoinositide 3-kinase (PI3K)/AKT (AKT serine/threonine kinase)/β-catenin axis, indicating the dual regulatory potential of microbial metabolites (**Figure 3B**) (36). Similarly, metformin treatment in FAP patients increases gut microbial diversity, decreases the abundance of *Ruminococcus* and elevates the level of *Lactobacillus*, accompanied by reduced polyp number and burden (37). Collectively, these findings indicate that gut microbiota cooperates with *APC* gene mutations to drive malignant transformation in FAP by secreting toxins, producing carcinogenic metabolites and regulating immune responses. Interventions targeting gut microbiota are expected to serve as a novel strategy for chemoprevention of FAP.

### 4.3 Bidirectional regulation between gut microbiota and metabolism in phenylketonuria

#### 4.3.1 Selective pressure of *PAH* mutations and low-phenylalanine diet on the gut microbiota

PKU is an autosomal recessive hereditary disease. Mutations in the phenylalanine hydroxylase (*PAH*) gene lead to loss or reduction of enzyme activity, resulting in abnormal accumulation of phenylalanine (Phe) in the body and subsequent neurological damage (38). The standard treatment for PKU is a lifelong low-Phe diet with strict restriction of natural protein intake. This special dietary pattern exerts profound and sustained selective pressure on gut microbiota. Studies have demonstrated that long-term low-Phe diet markedly alters the composition and diversity of gut microbiota in PKU patients. In particular, the abundances of butyrate-producing bacteria (genera affiliated with Lachnospiraceae and Ruminococcaceae) and *Lactobacillus*, which are closely associated with intestinal health, tend to decrease (39, 40). Such diet-induced microbial alterations are not unidirectional. Gut microbiota is capable of metabolizing Phe independently, thereby forming a bidirectional regulatory network with the host.

#### 4.3.2 Reverse modification of blood phenylalanine levels by microbial metabolic bypass pathways in PKU

Specific intestinal bacteria, including certain species of *Clostridium* and *Bacteroides*, can express enzymes such as Phe deaminase. They convert intestinal Phe into metabolites like phenylacetic acid and phenyllactic acid via deamination pathways (42, 43). These bacterial metabolic activities create a local “metabolic shunt” in the intestine, which catabolizes partial dietary and endogenous Phe and may in turn regulate serum Phe levels in the host. This hypothesis has been strongly validated by animal experiments. Studies have shown that after gut microbiota transplantation from healthy wild-type mice to PKU model mice, the intestinal Phe clearance capacity of recipient mice is enhanced, accompanied by a remarkable reduction in serum Phe concentration (41). These findings clearly indicate that healthy gut microbiota or its specific functional consortia may act as an “extra organ” for Phe metabolism and assist the host in alleviating metabolic burden (**Figure 3C**).

### 4.4 Research on gut microbiota in hereditary hemolytic anemias

Hereditary hemolytic anemias represent a group of genetic diseases caused by impaired hemoglobin synthesis or erythrocyte membrane defects. They affect tens of millions of people worldwide, with a higher prevalence in malaria-endemic regions. The core pathological manifestations include shortened erythrocyte lifespan and accelerated hemolysis, which consequently lead to anemia, jaundice, splenomegaly and multi-organ damage. Conventional treatments comprise blood transfusion, iron chelation and hematopoietic stem cell transplantation, which are accompanied by drawbacks such as infection risks, iron overload and donor shortage. In recent years, the roles of gut microbiota in various hematological diseases have been gradually elucidated. Gut microbiota may participate in the pathological progression of hemolytic anemias by regulating host immune responses, metabolite production and intestinal barrier function (122). For instance, patients with sickle cell disease frequently exhibit reduced gut microbial diversity and increased abundance of pathogenic bacteria (123), Iron overload in patients with thalassemia (Thal) can alter intestinal microecology and further exacerbate inflammation and oxidative stress (124). Accordingly, in-depth understanding of the interaction between gut microbiota and hereditary hemolytic anemias is of great significance for the development of novel therapeutic strategies.

#### 4.4.1 Alterations in diversity and abundance of gut microbiot

Multiple studies have consistently demonstrated that patients with hereditary hemolytic anemias, particularly those with sickle cell disease (SCD), exhibit markedly lower alpha diversity (e.g., Shannon index) of gut microbiota compared with healthy controls, indicating obvious intestinal microbial dysbiosis (125). At the phylum level, patients present profound structural changes in gut microbiota, including decreased relative abundance of *Firmicutes* and increased abundance of *Bacteroidetes* and *Proteobacteria*. The reduced F/B ratio is closely associated with chronic inflammation in the host (125). More distinct and crucial alterations are observed at the genus level. The abundances of SCFAs-producing beneficial genera, such as Faecalibacterium and Roseburia, are significantly decreased (126). In contrast, the levels of potential pathogens including *Escherichia* and *Enterococcus* are remarkably elevated (123). Such compositional shift characterized by depleted beneficial bacteria and enriched pathogens may disrupt intestinal barrier function and increase gut permeability, thereby facilitating systemic inflammation and accelerating disease progression (123, 126).

Distinct types of hereditary hemolytic anemias present unique gut microbiota profiles. In patients with Thal, long-term blood transfusion leads to systemic iron overload and alters the intestinal microenvironment. These patients exhibit significantly reduced bacterial diversity and distinct community structures compared with healthy individuals. For example, taxa associated with intestinal dysfunction, including *Clostridium* and members of Fusobacteriaceae, Enterobacteriaceae and Peptostreptococcaceae, are more prevalent in patients and correlate with elevated ferritin levels (127).

#### 4.4.2 Pathological mechanisms by which gut microbiota affects hereditary hemolytic anemias

Direct evidence remains limited, but the central role of gut microbiota in systemic inflammation, immune homeostasis, and metabolism supports reasonable inference. These diseases share chronic hemolysis, anemia, secondary iron overload, and persistent systemic inflammation, which the microbiota may modulate through multiple interrelated mechanisms (**Figure 4**).

**Figure 4:**
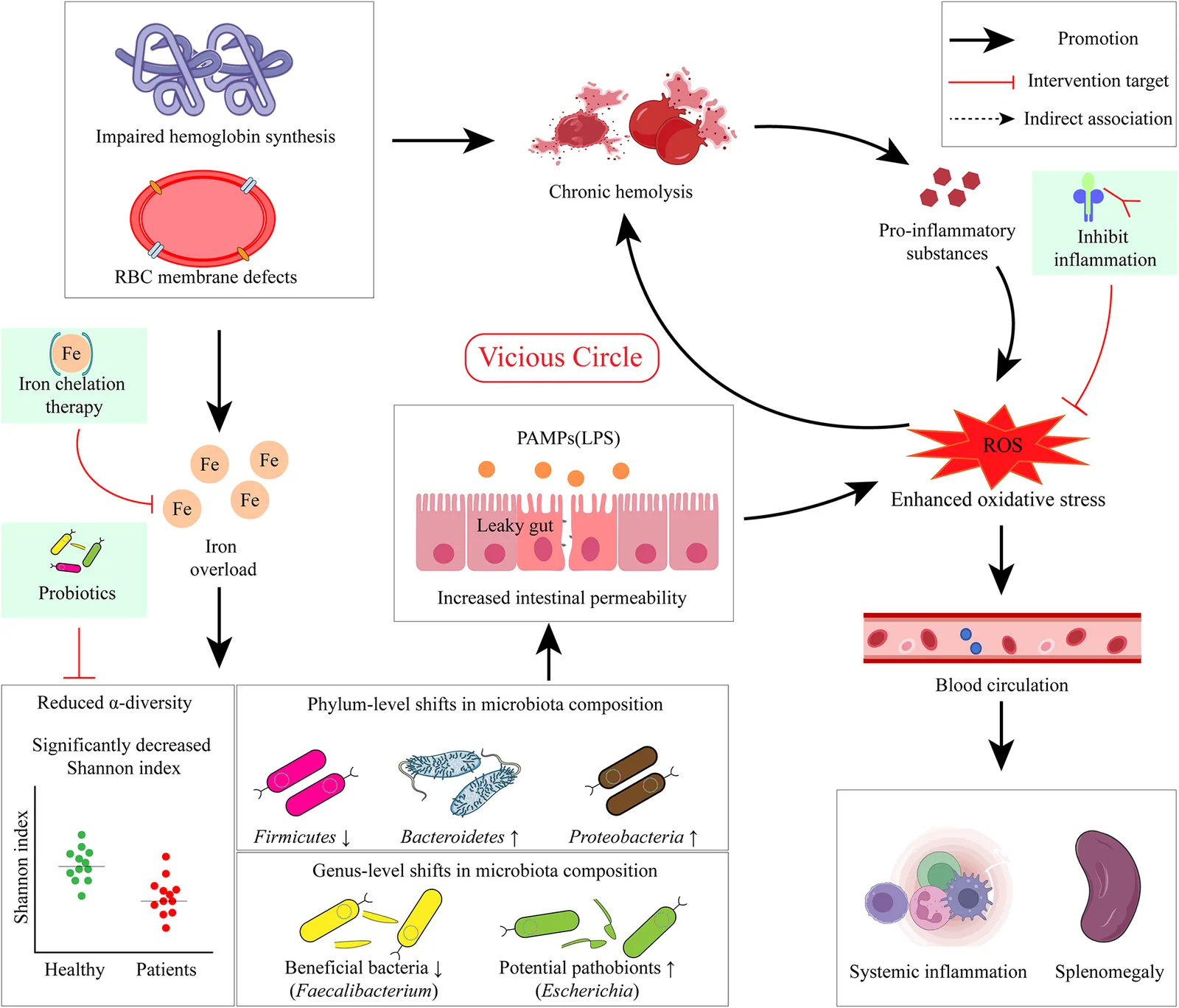
Vicious cycle linking gut dysbiosis, iron overload, and inflammation in hereditary hemolytic anemias. Impaired hemoglobin synthesis or erythrocyte membrane defects can trigger chronic hemolysis. Pro-inflammatory substances released by hemolysis cooperate with pathogen-associated molecular patterns (PAMPs, e.g., LPS) that leak into circulation through the compromised intestinal barrier, elevating ROS and exacerbating oxidative stress, which ultimately induces systemic inflammation and splenomegaly. Systemic inflammation in turn further promotes hemolysis, forming a vicious cycle. Meanwhile, secondary iron overload resulting from hemolysis drives gut microbiota structural dysbiosis, manifested by reduced microbial α-diversity (marked decrease in Shannon index), depletion of phylum *Firmicutes*, enrichment of phyla *Bacteroidetes* and *Proteobacteria* at the phylum level; at the genus level, beneficial taxa such as *Faecalibacterium* are decreased, whereas pathobionts including *Escherichia* are enriched. Gut dysbiosis further increases intestinal permeability, provokes leaky-gut phenotype and amplifies upstream inflammatory responses. Intervention targets in this pathway are annotated in the image: iron-chelating therapy corrects iron overload, and probiotic intervention modulates gut microbiota; suppression of inflammatory responses blocks oxidative-stress-mediated cascade injury.

Iron overload, a common complication, reshapes the intestinal microenvironment. Since iron supports the growth of Enterobacteriaceae, overload selectively promotes pathogenic bacteria while suppressing beneficial low-iron-requiring taxa (e.g., certain *Lactobacillus* and *Bifidobacterium*), leading to gut dysbiosis (128). In heterozygous β-Thal mice, iron overload induces dysbiosis, intestinal inflammation, and metabolic disorders (129). In Thal patients, iron overload also contributes to microbiota disturbance and cognitive impairment, suggesting probiotic strategies may help prevent gut–brain axis disorders.

Gut dysbiosis compromises intestinal epithelial barrier integrity, enabling bacterial endotoxin translocation into circulation and sustaining chronic low-grade inflammation (95, 130). Massive erythrocyte destruction releases pro-inflammatory and pro-oxidative hemoglobin and heme (131, 132); iron overload further promotes “leaky gut.” Translocation of intestinal biomolecules may exacerbate systemic inflammation and sepsis severity in Thal patients (133).

## 5 Gut microbiota modulates the progression of mitochondrial diseases via immune regulation

Mitochondrial diseases are a heterogeneous group of diseases caused by mutations in mitochondrial DNA (*mtDNA*) or nuclear DNA. Their core pathophysiology involves impaired oxidative phosphorylation and insufficient ATP production, which compromise the function of high-energy-demand organs such as the brain, muscles and heart (44). Common clinical syndromes include Leber’s hereditary optic neuropathy (LHON), mitochondrial encephalomyopathy with lactic acidosis and stroke-like episodes (MELAS), myoclonic epilepsy with ragged red fibers (MERRF), and Leigh syndrome (45). Despite remarkable advances in genetic diagnostics, such as the application of high-throughput sequencing and bioinformatic analysis that greatly improve the detection rate of disease-causing genes, current treatments for mitochondrial diseases remain largely symptomatic and supportive, including seizure control, diabetes management and metabolic disorder correction. Curative therapies targeting mitochondrial dysfunction fundamentally are still lacking (134). This therapeutic dilemma highlights the urgency to explore novel mechanisms underlying disease progression and identify new intervention targets. In recent years, gut microbiota, a critical regulator of host metabolism and immunity, has attracted increasing attention for its bidirectional crosstalk with mitochondrial function, namely the “microbiota-gut-mitochondria axis” (135).

### 5.1 Microbiota-induced inflammation and mitochondrial damage

Inflammatory cytokines generated via the TLR4/NF-κB pathway following gut microbiota dysbiosis can directly target mitochondria, inhibit the activities of respiratory chain complex I and IV, and trigger electron-transport-chain dysfunction (49). Meanwhile, inflammatory signals upregulate the expression of inducible nitric oxide synthase (iNOS) and trigger excessive nitric oxide (NO) production. NO reacts with superoxide leaked from the mitochondrial respiratory chain to generate highly oxidative peroxynitrite, which directly targets *mtDNA* and proteins and causes irreversible damage (47). In patients with LHON, elevated serum LPS levels are positively correlated with the severity of optic atrophy, suggesting that microbiota-induced systemic inflammation may accelerate the death of retinal ganglion cells carrying pathogenic *mtDNA* mutations (47). In addition, chronic inflammatory conditions activate the NOD-, LRR- and pyrin domain-containing protein 3 (NLRP3) inflammasome. Activation of this complex further reduces mitochondrial membrane potential and induces cytochrome c release, thereby amplifying mitochondrial damage signals and forming a vicious cycle between inflammation and mitochondrial dysfunction (136) (**Figure 5**).

**Figure 5:**
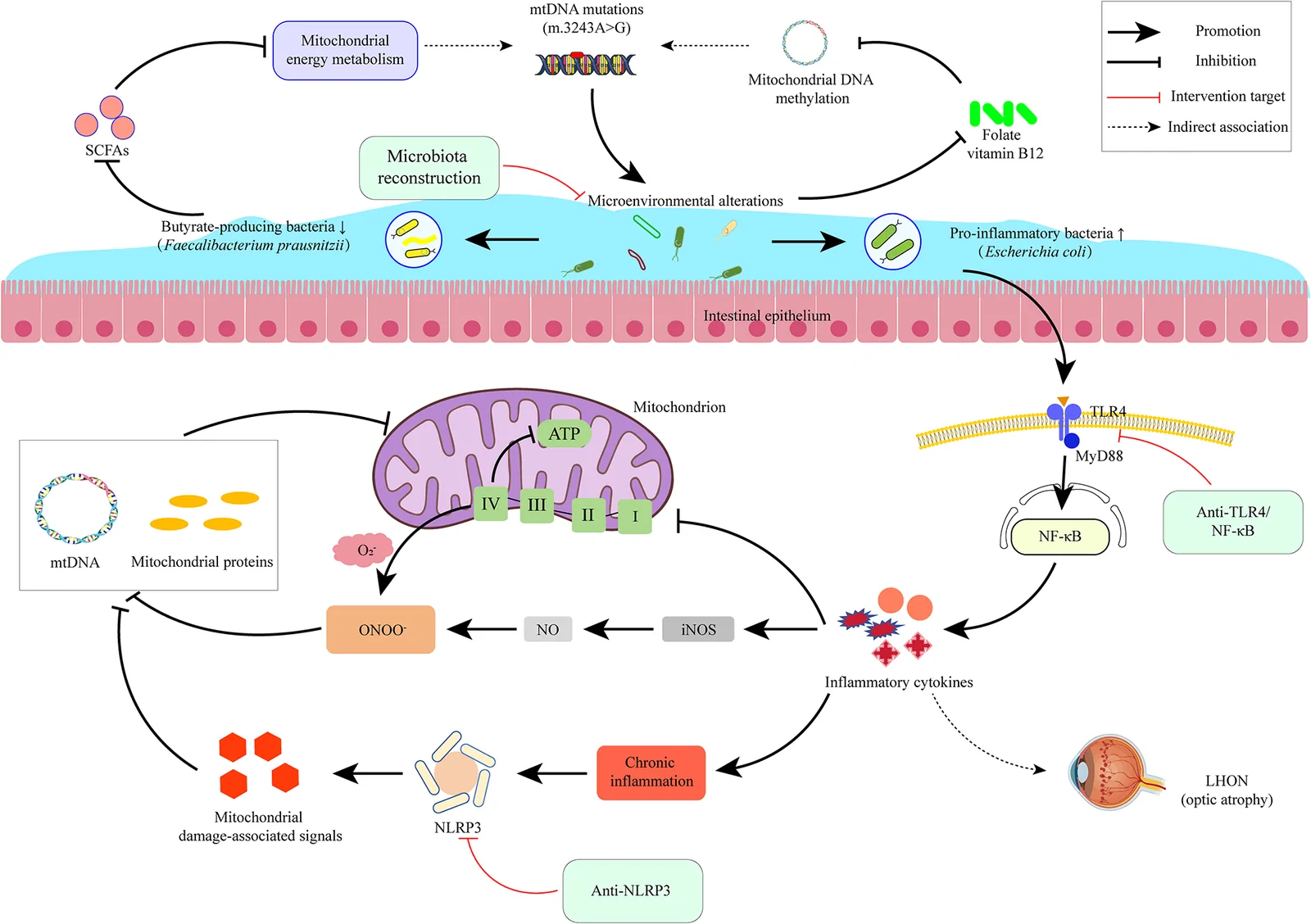
The gut dysbiosis–systemic inflammation–mitochondrial injury pathological axis. mtDNA mutations (e.g., m.3243A>G) can remodel the intestinal microenvironment and drive gut microbiota dysbiosis, characterized by reduced abundance of butyrate-producing bacteria (*Faecalibacterium prausnitzii*) and enrichment of pro-inflammatory bacteria (*Escherichia coli*). Dysregulated gut microbiota modulates mtDNA methylation via folate and vitamin B12, thereby indirectly participating in pathological processes linked to mtDNA mutations. Pathogenic components derived from dysbiotic microbiota activate the epithelial TLR4/NF-κB signaling axis and trigger the release of pro-inflammatory cytokines. Inflammation not only activates the NLRP3 inflammasome, but also upregulates iNOS to promote NO production. NO reacts with mitochondrial-derived superoxide anions to generate peroxynitrite (ONOO^-^), which further damages mitochondrial complex I and IV, leading to impaired ATP synthesis and massive release of mitochondrial damage-associated molecular signals, aggravating mitochondrial injury. Mitochondrial-derived damage signals in turn amplify inflammatory responses, forming a vicious cycle of inflammation-mitochondrial damage. Ultimately, these pathological events may induce LHON (Leber’s hereditary optic neuropathy). Intervention targets: reconstruction of gut microbiota, inhibition of the TLR4/NF-κB pathway, and NLRP3 inflammasome suppression. Multi-target interventions hold promise for breaking this pathological loop and provide adjunctive therapeutic strategies for mitochondrial diseases. This schematic incorporates the following third-party icons, reproduced without modification: the "plasmid-s-insert-dna" icon by DBCLS (https://togotv.dbcls.jp/en/pics.html is licensed under CC-BY 4.0 Unported https://creativecommons.org/licenses/by/4.0/) and the "dna-nucleotides-ribbon" icon by Servier (https://smart.servier.com/ is licensed under CC-BY 3.0 Unported https://creativecommons.org/licenses/by/3.0/).

### 5.2 Correlation between gut microbiota composition and *mtDNA* mutation load

Different types of *mtDNA* mutations may selectively shape gut microbiota composition by altering the host metabolic microenvironment. For instance, patients with mitochondrial encephalomyopathy carrying the *m.3243A>G* mutation exhibit a marked reduction in butyrate-producing bacteria such as *Faecalibacterium prausnitzii*, accompanied by an increase in pro-inflammatory bacteria including *E. coli (*46). Such gut dysbiosis may modulate *mtDNA* methylation by regulating the levels of host methyl donors such as folate and vitamin B12, thereby altering the expression and heteroplasmy of mutant genes (48). Animal experiments further verify that gut microbiota transplantation from mice with mitochondrial myopathy into germ-free mice leads to decreased mitochondrial respiratory chain activity and elevated oxidative stress in recipients, indicating that dysbiotic microbiota can directly transmit the phenotype of mitochondrial dysfunction (137). In addition, the association between microbial composition and *mtDNA* mutation load is reflected in the crosstalk of metabolic pathways. Specifically, the depletion of butyrate-producing bacteria reduces the concentration of SCFAs, which serve as crucial substrates for mitochondrial energy metabolism (138). Accordingly, specific *mtDNA* mutations can remodel the intestinal microenvironment and drive the shift of microbiota toward a pro-inflammatory and metabolically imbalanced profile. This process forms a vicious cycle and further exacerbates mitochondrial dysfunction.

## 6 Regulatory roles of gut microbiota in polygenic hereditary diseases

### 6.1 Characteristics and functional alterations of gut dysbiosis in IBD

#### 6.1.1 Alterations in gut microbiota composition, diversity and metabolic functions

A marked reduction in microbial diversity and compositional imbalance are among the hallmark features of gut microbiota in patients with IBD. Compared with healthy individuals, patients with IBD exhibit significantly decreased alpha diversity of intestinal microbes, and their beta diversity is distinctly separated from that of healthy controls (52, 53). This dysbiosis is characterized by reduced abundance of beneficial bacteria and an increased proportion of potential pathogens. Multiple studies have consistently reported decreased abundances of *Firmicutes* and *Bacteroidetes*, as well as remarkable elevation of *Proteobacteria* and *Actinobacteria* in the gut of IBD patients (139). At the genus level, anti-inflammatory bacteria that produce SCFAs including butyrate, such as *Faecalibacterium*, *Roseburia* and *Ruminococcus*, are markedly depleted, whereas pro-inflammatory *E. coli* and other related taxa are significantly enriched (140, 141). A cross-cohort integrative metagenomic–metabolomic analysis (CCIA) encompassing nine independent cohorts (n = 1,363 IBD patients) identified a stable diagnostic marker panel comprising 31 species, 25 functional genes, and 13 metabolites; the integrated model achieved an area under the receiver operating characteristic curve (AUROC) of 0.98 and revealed aminoacyl-tRNA biosynthesis and two-component signal transduction systems as core microbiota functional pathways in IBD (142), significantly outperforming single-omics or single-cohort studies. Mucosa-associated microbiota in the ileum of Crohn’s disease (CD) patients shows prominent enrichment of adherent-invasive *Escherichia coli* (AIEC) and *Fusobacterium (*54). By contrast, depletion of *Roseburia* and *Faecalibacterium prausnitzii* is commonly observed in ulcerative colitis (UC) patients (141). Furthermore, gut dysbiosis is not limited to fecal samples. Changes in mucosa-associated microbiota are closely correlated with disease activity and inflammatory sites, indicating that local microecological imbalance plays a pivotal role in the pathogenesis of IBD (143).

Metabolic pathways of gut microbiota are profoundly remodeled in IBD patients, and such functional alterations are critical to disease progression. First, the reduction of SCFA-producing taxa (e.g., *Faecalibacterium*), with consequent impairment of intestinal barrier function and anti-inflammatory signaling, constitutes one of the hallmark features of IBD (56). Second, bile acid metabolism is also disrupted. The microbiota-mediated conversion of primary bile acids to secondary bile acids is impaired, resulting in dysregulated signaling of farnesoid X receptor (FXR) and G-protein coupled receptor (TGR5), which further exacerbates inflammatory responses (144, 145). Reduced production of indole metabolites (e.g., indole-3-acetic acid) by gut microbiota weakens aryl hydrocarbon receptor (AhR)-mediated immunomodulation and aggravates intestinal inflammation (144). These metabolic changes do not occur in isolation but interact with each other, collectively forming a complex network of metabolic diseases in IBD. For instance, butyrate deficiency not only directly damages the intestinal epithelial barrier, but also exacerbates inflammation by regulating immune cell differentiation (146). Accordingly, targeting these metabolic pathways, such as SCFA supplementation or bile acid metabolism modulation, may serve as novel therapeutic strategies for IBD.

#### 6.1.2 Modification of IBD disease activity and drug response heterogeneity by gut dysbiosis

IBD patients exhibit markedly abnormal spatial distribution of the microbiota: in healthy individuals, commensal bacteria predominantly reside within the intestinal lumen, maintaining a distance from the epithelium, whereas in IBD, thinning of the mucus layer and disruption of the intestinal barrier facilitate microbial translocation toward the mucosal surface, where dense biofilms form and establish direct contact with immune cells—a process central to disease pathogenesis. Among these, AIEC is the most extensively studied; this pathobiont utilizes flagella, type I fimbriae, and other adhesins to bind intestinal epithelial carcinoembryonic antigen-related cell adhesion molecules (CEACAMs), thereby accomplishing colonization and invasion (57, 58). Following internalization into macrophages, AIEC proliferates robustly, inducing the release of pro-inflammatory cytokines including interleukin-1β (IL-1β) and TNF-α, thereby exacerbating intestinal inflammation (59, 60). Moreover, bacteria within biofilms can synchronize virulence expression through quorum sensing, further amplifying pathogenic capacity. Microbial spatial ectopia also represents a critical contributor to the recurrent and chronic course of IBD; consequently, biofilm disruption and bacterial adhesion blockade constitute potential novel therapeutic targets (147).

The gut microbiota orchestrates intestinal inflammatory homeostasis through dual pathways of innate and adaptive immunity. At the innate immune level, microbial pathogen-associated molecular patterns (PAMPs)—including LPS, peptidoglycan, and flagellin—are recognized by pattern recognition receptors such as Toll-like receptors (TLRs) and NOD-like receptors (NLRs) expressed on epithelial cells and within the lamina propria (55). Specifically, LPS activates TLR4, peptidoglycan activates Toll-like receptor 2 (TLR2), and muramyl dipeptide (MDP) activates nucleotide-binding oligomerization domain-containing protein 2 (NOD2), collectively initiating the NF-κB and mitogen-activated protein kinase (MAPK) pathways to regulate the secretion of diverse inflammatory mediators (148). Loss-of-function mutations at the core CD susceptibility locus NOD2 impair MDP recognition, diminish α-defensin synthesis, and markedly increase the risk of pathogen translocation, thereby perpetuating intestinal inflammation (51) (**Figure 6**). At the adaptive immune level, the microbiota governs the Th17/Treg differentiation balance and modulates B cell secretion of secretory IgA (sIgA). Segmented filamentous bacteria (SFB) induce epithelial secretion of serum amyloid A (SAA), thereby driving Th17 cell differentiation (55). These cells possess dual attributes of barrier protection and pathogenicity; in genetically susceptible individuals, hyperactivated Th17 cells release abundant interleukin-17 (IL-17) and interleukin-22 (IL-22), mediating pathological intestinal mucosal damage (149). In contrast, specific strains of *Clostridium* and *Bacteroides* promote Foxp3+ Treg proliferation through butyrate and other SCFAs (145). Tregs, in turn, secrete IL-10 and transforming growth factor-β (TGF-β) to suppress effector T cells, thereby preserving intestinal immune tolerance (150). Concurrently, the microbiota induces IgA+ plasma cell differentiation through TLR signaling and metabolites such as retinoic acid (151), secretory IgA encases commensal bacteria, preventing their direct contact with the epithelium and thereby maintaining host–microbial symbiotic homeostasis (151) (**Figure 6**).

**Figure 6:**
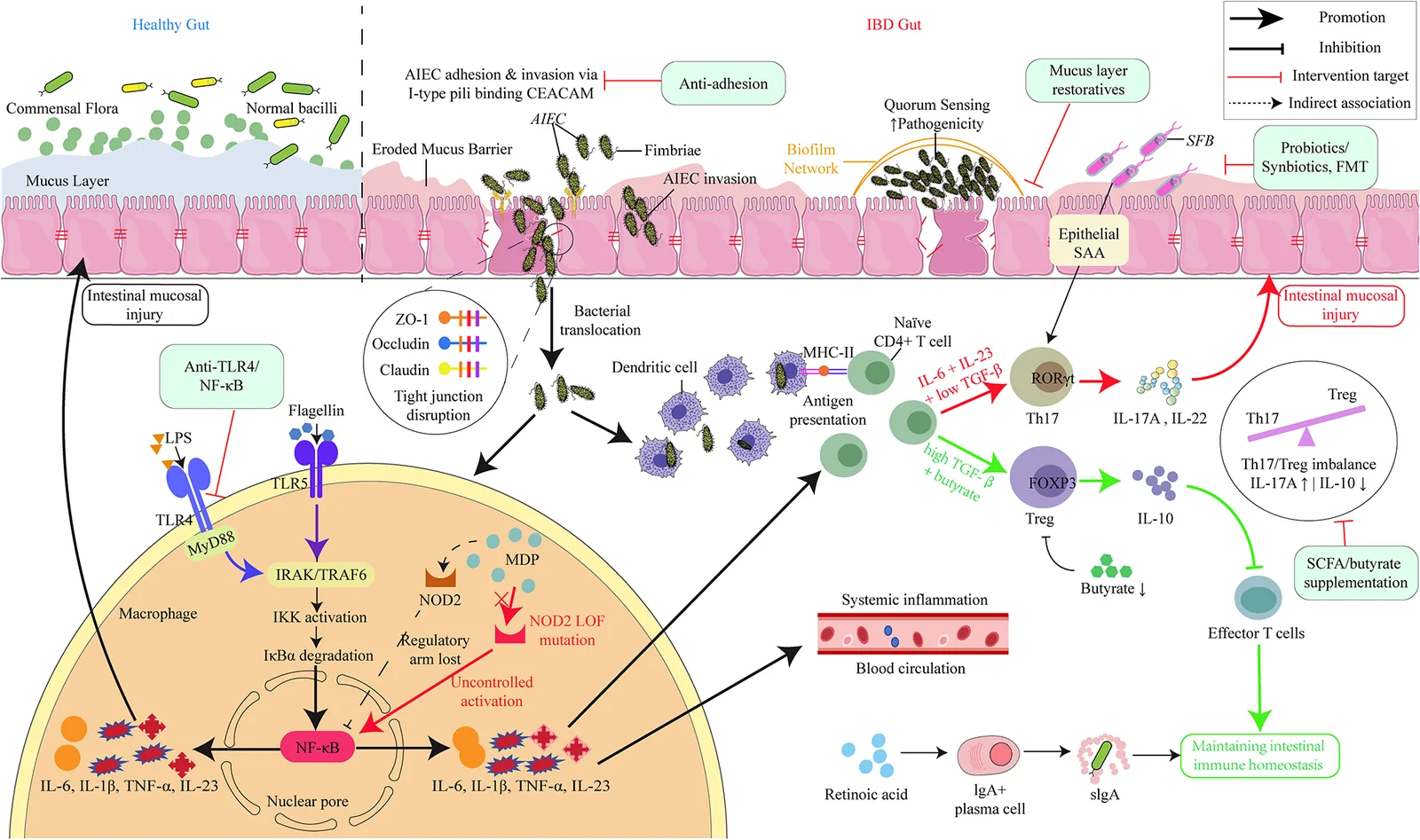
Mechanism by which abnormal microbiota spatial distribution triggers immune cascade responses in IBD. In IBD, the inner mucus layer is degraded and thinned (mucus degradation), which removes the spatial confinement of gut microbiota. Biofilms form on the epithelial surface, and adherent-invasive *Escherichia coli* (AIEC) adhere to and invade intestinal epithelial cells (AIEC adhesion and invasion). Disruption of tight-junction proteins (ZO-1, occludin, claudin) increases intestinal barrier permeability, enabling bacterial translocation across the epithelium into the lamina propria. Translocated bacteria and their PAMPs are recognized by pattern-recognition receptors, including LPS-TLR4, flagellin-TLR5, and MDP-NOD2. TLR4 activation initiates the intracellular MyD88/IRAK/TRAF6 signaling cascade, leading to IKK complex activation, IκBα degradation and NF-κB nuclear translocation, which drives transcription of pro-inflammatory cytokines (TNF-α, IL-1β, IL-6 and IL-23). Loss-of-function NOD2 mutations (e.g., L1007fs) in Crohn’s disease abolish the regulatory branch that normally suppresses NF-κB activation via IRF4/IL-10, resulting in unrestrained amplification of inflammatory signaling. Dendritic cells (DCs) take up bacteria and present antigens to naive CD4^+^ T cells. Macrophage-derived IL-6 and IL-23, together with low concentrations of TGF-β, polarize naive CD4^+^ T cells toward pro-inflammatory RORγt^+^ Th17 cells that secrete IL-17A and IL-22. By contrast, high levels of TGF-β combined with butyrate promote the differentiation of FOXP3^+^ Tregs with IL-10 secretion. This balance shifts toward Th17 dominance in IBD (Th17/Treg imbalance; elevated IL-17A/reduced IL-10). SFB induce epithelial SAA secretion to promote Th17-cell differentiation. Metabolites such as retinoic acid drive the differentiation of IgA^+^ plasma cells; secretory IgA coats commensal bacteria to maintain host-commensal homeostasis. Intervention-target layer: block AIEC adhesion/disrupt biofilms; inhibit innate immunity by targeting the TLR4/NF-κB pathway; restore Th17/Treg balance via butyrate/SCFA supplementation; reconstruct gut microbiota using probiotics or fecal microbiota transplantation; repair the mucus-layer barrier. This schematic incorporates the following third-party icons, reproduced without modification: the "cell-1" icon by Servier (https://smart.servier.com/ is licensed under CC-BY 3.0 Unported https://creativecommons.org/licenses/by/3.0/) and the "mast_cell_1" icon by Sebastian-Reinig is licensed under CC-BY 4.0 Unported (https://creativecommons.org/licenses/by/4.0/).

### 6.2 Gut microbiota profiles and dysbiosis in patients with ASD

ASD is a neurodevelopmental disease characterized by deficits in social communication, repetitive stereotyped behaviors and restricted interests, with a markedly increasing global incidence (61). In recent years, accumulating evidence has demonstrated that patients with ASD frequently present prominent gastrointestinal symptoms including constipation, diarrhea and abdominal pain, whose prevalence is up to four times higher than that in the general population (61). This phenomenon strongly suggests that intestinal dysfunction has an intrinsic bidirectional correlation with neurodevelopmental abnormalities, rather than being merely a comorbidity (152). The emerging concept of the gut-brain axis provides a crucial framework for understanding this complex relationship. This axis encompasses multiple pathways including gut microbiota, intestinal barrier, immune system, endocrine system and vagus nerve, forming an intricate bidirectional signaling network between the gastrointestinal tract and the brain (153, 154). Dysregulation of this network is recognized as a major environmental factor contributing to or exacerbating the core behavioral symptoms of ASD (154).

#### 6.2.1 Alterations in microbial composition and diversity

Significant differences in both alpha and beta diversity of gut microbiota have been observed between children with ASD and neurotypical children, while inconsistencies exist among published findings. Several studies have reported that the alpha diversity of gut microbiota (e.g., Shannon index) is markedly lower in children with ASD than in healthy controls, and such reduction is correlated with ASD severity (65). Nevertheless, large-scale studies have detected no significant differences in alpha and beta diversity among children with ASD, their unaffected siblings and healthy controls, suggesting that alterations in microbial communities may be individual-specific (66). Characteristic changes in the abundance of specific microbes are commonly found in children with ASD. Certain species of *Clostridium*, including *Clostridium difficile* and *Clostridium perfringens*, are significantly enriched in fecal samples from ASD children compared with neurotypical counterparts. Some *Clostridium* species such as *Clostridium bolteae* are exclusively detected in children with ASD (64). An imbalanced ratio of *Bacteroidetes* to *Firmicutes*, together with depletion of beneficial bacteria including *Bifidobacterium* and *Lactobacillus*, are also common features (155). These compositional changes of gut microbiota are closely associated with intestinal inflammation and metabolic diseases. For instance, reduced abundance of butyrate-producing bacteria such as *Faecalibacterium* and *Agathobacter* in children with ASD may lower SCFA levels, thereby impairing intestinal barrier function and immune regulation (62).

#### 6.2.2 Correlation between gut dysbiosis and the severity of ASD

Multiple cross-sectional studies using 16S rRNA gene sequencing or metagenomic analysis have identified statistically significant associations between specific gut microbial profiles and behavioral scores of ASD. For example, a study on monozygotic twins revealed no significant difference in overall microbial diversity between patients with mild and severe ASD. However, *Porphyromonas* was markedly enriched in the severe ASD group, accompanied by downregulated expression of genes related to carbohydrate metabolism and stress response, indicating a link between microbial functional disturbances and ASD severity (156). At the genus level, the abundance of certain *Clostridium* species was positively correlated with ASD symptom severity, whereas the abundance of *Akkermansia muciniphila* was associated with improved social behaviors (63). In addition, longitudinal studies have preliminarily indicated that early gut dysbiosis may precede or coincide with the onset of behavioral symptoms of ASD. For instance, a study identified three microbial markers including *Faecalitalea*, *Caproiciproducens* and *Collinsella* via machine learning models, which could distinguish ASD patients from healthy controls with an area under the curve (AUC) of 0.94. This finding suggests that gut microbiota may serve as biomarkers for ASD diagnosis and disease progression (157). Collectively, these findings prove that specific compositional patterns of gut microbiota are closely correlated with ASD severity, laying an important foundation for elucidating the pathogenesis of ASD and developing microbiota-based therapeutic strategies.

#### 6.2.3 Intestinal barrier dysfunction and immune system activation

Patients with ASD frequently suffer from gastrointestinal symptoms. The “leaky gut” phenotype is regarded as one of the key links connecting intestinal abnormalities to core ASD symptoms. Increased intestinal permeability in individuals with ASD allows incompletely digested food macromolecules (such as gluten and casein peptides) and bacterial endotoxins including LPS to cross the intestinal epithelial barrier and enter the bloodstream (67). Such barrier impairment is mainly caused by the downregulated expression or dysfunction of tight junction proteins that maintain intercellular integrity of intestinal epithelial cells, including zonula occludens-1 (ZO-1), occludin and the claudin family (68). Both valproic acid (VPA)–induced and maternal separation ASD-related animal models have demonstrated downregulation of tight junction–encoding gene expression and elevated circulating LPS, substantiating the molecular basis of leaky gut (69, 70). Circulating substances such as LPS can trigger a state of systemic low-grade inflammation via the TLR4/NF-κB pathway, thereby impairing distal organ functions (158). Notably, although zonulin is a crucial regulator of tight junctions, a study on Italian ASD patients failed to identify a significant association between its coding gene haptoglobin 2 (HP2) and ASD, suggesting that leaky gut may involve more complicated molecular mechanisms (159). Dysregulated zinc ion (Zn²^+^) homeostasis is also implicated in impaired intestinal barrier integrity. Zn²^+^ deficiency exacerbates intestinal inflammation and leaky gut (160). Collectively, these findings illustrate the multifactorial molecular basis of leaky gut in ASD and provide important clues for elucidating the underlying pathogenesis (**Figure 7A**).

**Figure 7:**
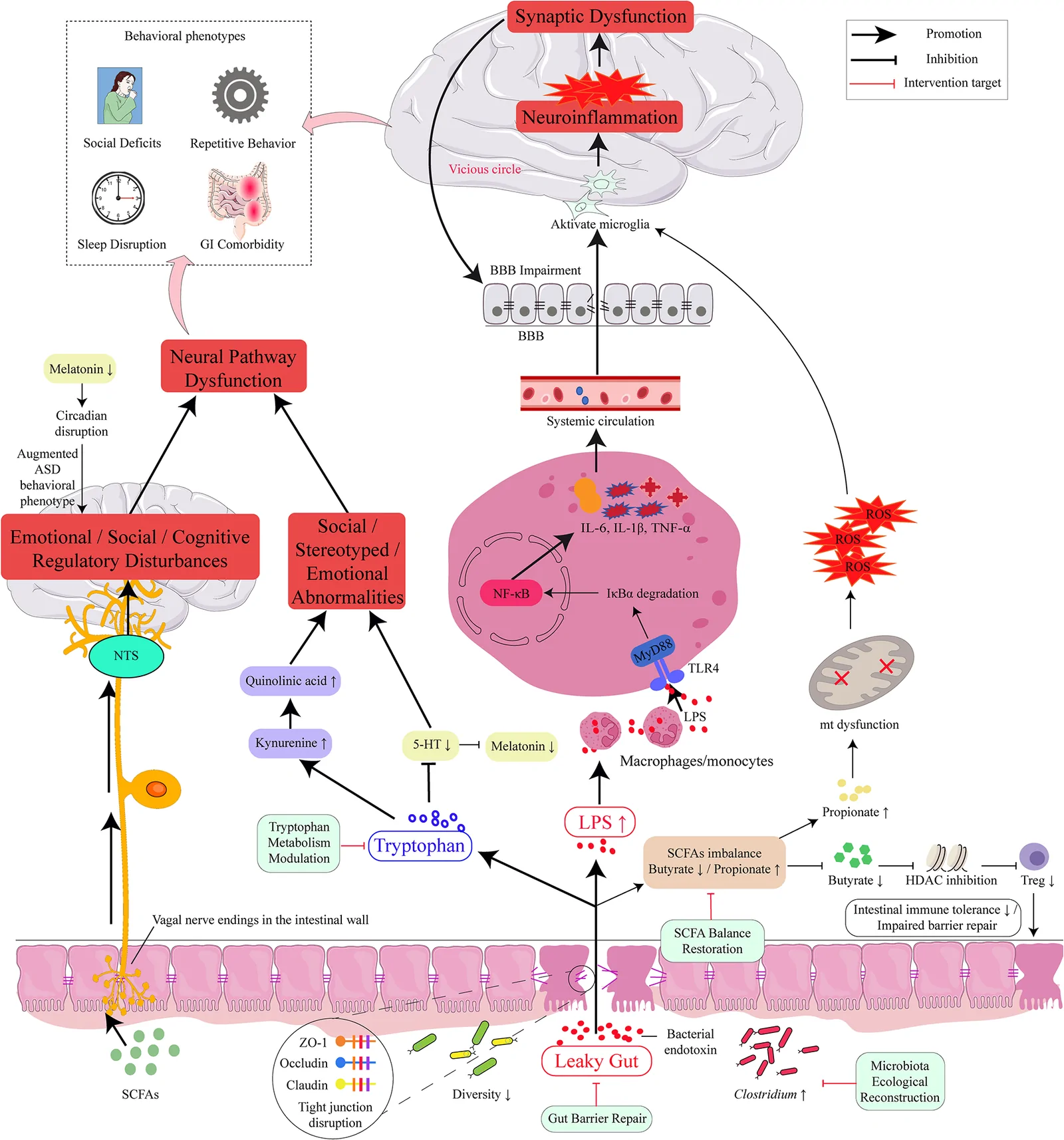
Multi-pathway mechanisms of gut–brain axis interactions in ASD. Patients with ASD exhibit reduced gut microbiota diversity, accompanied by expansion of pro-inflammatory taxa such as *Clostridium*, whose abundance correlates with symptom severity. Impairment of intestinal epithelial tight-junction proteins (ZO-1, occludin, claudin) gives rise to “leaky gut”, permitting microbial products to translocate across the barrier into the circulation. Circulating LPS activates the TLR4/MyD88/NF-κB cascade in peripheral macrophages, driving the release of TNF-α, IL-6 and IL-1β into the bloodstream. These inflammatory factors cross the compromised BBB and trigger microglial activation as well as astrogliosis, ultimately resulting in synaptic dysfunction. A vicious cycle is established within the brain: neuroinflammation-BBB disruption-further infiltration of peripheral inflammatory mediators. Elevated propionic acid exacerbates neuronal injury in this cascade by inducing mitochondrial dysfunction and excessive ROS production. By contrast, butyrate deficiency creates a local positive-feedback loop that aggravates intestinal leakage through attenuated HDAC inhibition, impaired Treg differentiation and defective barrier repair. Tryptophan metabolism is shifted toward neurotoxic quinolinic acid production; meanwhile, 5-HT synthesis is suppressed, which further reduces melatonin levels, thereby causing circadian rhythm disruption and sleep disturbances and worsening ASD-related behavioral phenotypes. Microbial metabolites stimulate vagal afferent terminals in the intestinal wall, which project via the nucleus tractus solitarius to the limbic system and prefrontal cortex, contributing to aberrations in emotion, social interaction and cognition. The behavioral outputs encompass four core ASD phenotypes: social deficits, stereotyped behaviors, sleep disturbance and gastrointestinal comorbidities. Intervention targets: Repair intestinal barrier function (restore tight-junction integrity and block LPS translocation); Rebalance SCFA homeostasis (supplement butyrate/butyrate-producing bacteria and restrict propionic acid); Modulate tryptophan metabolism (inhibit the kynurenine pathway and recover serotonin synthesis); Reconstruct gut microbial ecology (probiotics/synbiotics/FMT targeting common upstream drivers). This schematic incorporates the following third-party icons, reproduced without modification: the "mitochondria" icon by jaiganesh (https://github.com/jaiganeshjg is licensed under CC0 https://creativecommons.org/publicdomain/zero/1.0/).

Leaky gut resulting from intestinal barrier dysfunction not only induces local intestinal inflammation, but also triggers systemic and central nervous system inflammation via immune activation. This process is recognized as a core pathological link between intestinal abnormalities and behavioral symptoms of ASD. Elevated levels of pro-inflammatory cytokines, including in IL-6, IL-1β and TNF-α, are frequently detected in the peripheral blood of ASD patients (161, 162). These circulating inflammatory cytokines can penetrate the blood-brain barrier (BBB) or activate cerebral vascular endothelial cells, thereby exerting impacts on the central nervous system (71). Cerebrospinal fluid analysis of adult patients with ASD revealed significantly higher total protein levels and albumin quotient compared with controls, indicating impaired BBB function (163). Region-specific alterations in the brain endocannabinoid system (eCBome) in fragile X mental retardation 1 (*Fmr1-/y*) mouse models further confirm the presence of neuroinflammation. For instance, the anti-inflammatory factor dihomo-gamma-linolenic acid (DGLA) is decreased in the hippocampus, whereas the pro-inflammatory mediator 12-hydroxylheptadecatrienoic acid is increased, the latter serving as a marker of microglial activation (164). Neuroinflammation directly impairs neuronal function, alters synaptic plasticity and disturbs neurotransmitter systems. For example, astrocytes exhibit aberrant glutamate metabolism in ASD, which directly disrupts neuronal network activity. Meanwhile, microglia show defective synaptic pruning, a critical developmental process for the refinement of neuronal connections (165, 166). In addition, downregulation of the tight junction protein claudin-5 in the BBB is recognized as one of the key mechanisms driving increased BBB permeability and exacerbating neuroinflammation (167). These findings demonstrate that a complete pathological cascade runs from intestinal barrier dysfunction to systemic inflammation and subsequent neuroinflammation, ultimately leading to neural and behavioral abnormalities associated with ASD.

#### 6.2.4 ASD-specific disturbances in SCFAs, tryptophan metabolism, and gut–brain axis pathways

The general signaling mechanisms of SCFAs, tryptophan metabolism, and gut–brain axis communication have been detailed in Sections 1.4.2, 1.4.3, and 5.2.3. Here we integrate ASD-specific evidence linking these pathways to pathophysiology (**Figures 7B–D**).

First, SCFA imbalance is consistently observed in ASD, with reduced butyrate and relatively elevated propionate; probiotic/fructooligosaccharide interventions that elevate butyrate correlate with symptomatic improvement, whereas excess propionate induces ASD-like behaviors and neuroinflammation in animal models (168–170). Second, tryptophan metabolism is skewed toward the kynurenine pathway at the expense of serotonin and melatonin synthesis (171, 172); elevated platelet serotonin and altered plasma 5-hydroxyindoleacetic acid (5-HIAA) correlate with behavioral symptom severity in children with ASD (173, 174), and microbial p-cresol further disrupts this pathway (174–176). Third, the vagal, neuroendocrine (GLP-1/PYY), and immune-inflammatory routes of gut–brain communication are all functionally impaired in ASD, contributing to social deficits, anxiety, and feeding disturbances (115, 152); multi-omics evidence additionally identifies bacterial products and neurotransmitter metabolites capable of crossing the blood–brain barrier, providing human molecular support linking intestinal dysbiosis to neuroinflammation (177–179).

Together, these metabolite shifts and gut–brain pathway impairments reinforce the barrier-inflammation-neuroinflammation cascade described in Section 5.2.3, forming a multi-pathway network underlying gut–brain axis dysfunction in ASD (**Figure 7**).

### 6.3 Pathogenic mechanisms linking gut dysbiosis to diabetes and obesity

#### 6.3.1 Association between altered microbiota composition and metabolic phenotypes

Patients with obesity and type 2 diabetes mellitus (T2DM) exhibit characteristic compositional alterations in gut microbiota, which are closely associated with host metabolic phenotypes. Numerous studies have confirmed that, compared with healthy individuals, patients with obesity and T2DM show an increased relative abundance of Firmicutes and a decreased abundance of Bacteroidetes in the gut, resulting in a markedly elevated Firmicutes/Bacteroidetes (F/B) ratio (73, 74). Such structural dysbiosis is generally accompanied by reduced abundance of butyrate-producing bacteria such as *Roseburia* and *Faecalibacterium*, while LPS-producing Proteobacteria and TMAO-producing *Clostridium* are relatively enriched (75). LPS translocation activates TLR4/NF-κB, leading to hepatic and adipose tissue insulin resistance and amplifying both local and systemic inflammation. In addition, reduced microbial diversity is recognized as an independent risk factor for metabolic diseases. Individuals with low microbiota diversity are more susceptible to weight gain, dyslipidemia and elevated inflammatory markers (76, 77). These findings indicate that specific compositional shifts of gut microbiota are not merely epiphenomena of metabolic diseases, but likely serve as key drivers of disease onset and progression.

#### 6.3.2 Mechanisms of insulin resistance mediated by gut microbial metabolites

Metabolites derived from gut microbiota play a central role in the development and progression of insulin resistance. Multi-omics analyses have revealed that the gut microbiota of insulin-resistant individuals exhibits enhanced carbohydrate metabolism, leading to accumulation of monosaccharides such as fructose and galactose in feces; these unabsorbed sugars promote insulin resistance through activation of hepatic lipogenic pathways (78). Furthermore, under high-fat dietary conditions, microbiota-derived branched-chain fatty acids can activate the mechanistic target of rapamycin complex 1 (mTORC1) signaling pathway, inducing serine phosphorylation of insulin receptor substrate-1 (IRS-1), thereby directly impairing insulin signal transduction (79). Meanwhile, excessive activation of FXR promotes hepatic gluconeogenesis and exacerbates hyperglycemia (80). The combined dysregulation of these metabolites forms a complex molecular network linking gut dysbiosis to insulin resistance.

#### 6.3.3 Roles of the microbiota-gut-brain axis in metabolic regulation

The microbiota-gut-brain axis is a vital pathway for gut microbiota to regulate host metabolism. It modulates the central nervous system via neural, endocrine and immune routes, thereby controlling appetite and energy expenditure. Microbial metabolites such as SCFAs can activate vagal afferent fibers or directly enter the bloodstream to act on G-protein-coupled receptor 41 (GPR41) in the hypothalamic arcuate nucleus and suppress appetite (180). Gut dysbiosis also causes aberrant synthesis of neurotransmitters including serotonin and γ-aminobutyric acid, which disturbs mood and feeding behavior. Obese individuals frequently present microbiota-mediated depressive and anxiety-like behaviors (181). Animal studies have provided solid evidence that microbiota transplantation from obese mice leads to increased appetite and reduced energy expenditure in recipient mice, which directly verifies the capacity of gut microbiota to regulate host metabolism through the gut-brain axis (73). In addition, high-fat or high-sugar diets alter microbial composition, activate the TLR4 inflammatory pathway and induce neuroinflammation, which further disrupts hypothalamic regulation of energy homeostasis (180). These findings highlight the central role of the microbiota-gut-brain axis in metabolic regulation (**Figure 8**).

**Figure 8:**
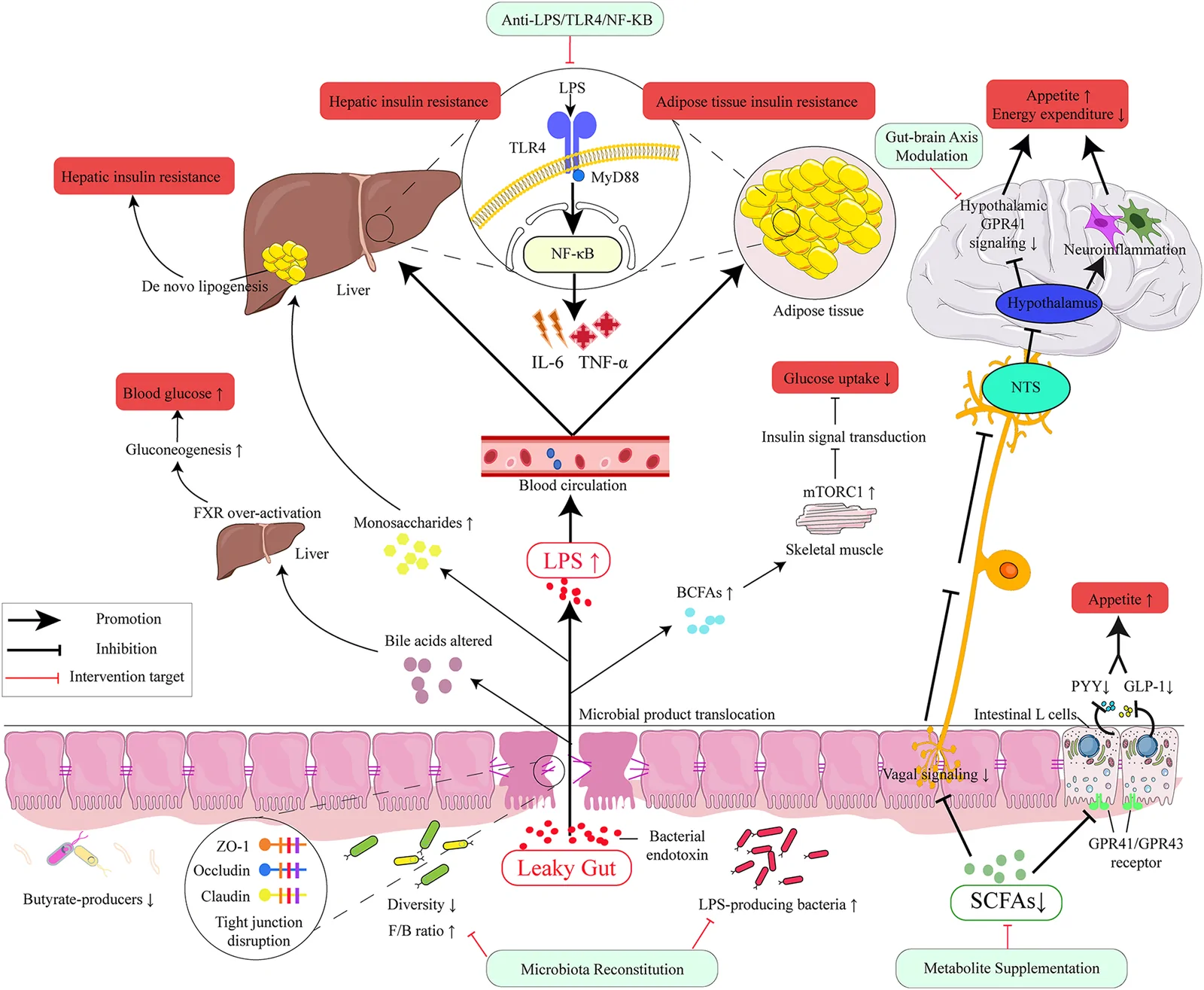
Integrated model of gut dysbiosis influencing obesity and T2DM. Patients with obesity and T2DM exhibit reduced gut microbiota diversity, an elevated *Firmicutes*/*Bacteroidetes* ratio, expansion of LPS-producing bacteria, and depletion of butyrate-producing bacteria such as *Faecalibacterium* and *Roseburia*. Disruption of intestinal epithelial tight-junction proteins (ZO-1, occludin, claudin) leads to “metabolic endotoxemia”, allowing microbial products to cross the barrier into the circulation. Via systemic circulation, LPS is delivered to the liver and adipose tissues, where it activates the TLR4/MyD88/NF-κB cascade and triggers the release of pro-inflammatory cytokines (TNF-α, IL-6), resulting in hepatic and adipose-tissue insulin resistance, respectively. Adipose tissue concurrently displays adipocyte hypertrophy, amplifying local and systemic inflammation. Increased monosaccharides are transported to the liver to drive de-novo lipogenesis, contributing to hepatic steatosis and insulin resistance. Elevated branched-chain fatty acids reach skeletal muscle and activate mTORC1, impairing insulin signal transduction and reducing glucose uptake. The altered bile-acid profile activates hepatic FXR, which excessively stimulates hepatic gluconeogenesis and elevates blood glucose. Regarding the gut-brain axis: decreased butyrate acts on intestinal endocrine L-cells to reduce secretion of appetite-suppressing hormones GLP-1 and PYY, and attenuates vagal afferent stimulation. Such attenuated signals, together with microbiota-driven neuroinflammation, are transmitted to the hypothalamus, weakening GPR41 signaling and inducing neuroinflammation, thereby disrupting energy-homeostasis regulation, increasing appetite and lowering energy expenditure. Intervention targets: Microbiota ecological restoration: probiotics/synbiotics/FMT to correct the F/B ratio and restore microbial diversity; Metabolite compensation: supplementation of SCFAs/butyrate to restore barrier function and inhibit LPS translocation; Inflammatory-pathway blockade: anti-LPS/TLR4/NF-κB strategies to rescue IRS-1 signaling; Gut-brain-axis modulation: targeting GPR41 and neurotransmitters to recover appetite and energy homeostasis. This schematic incorporates the following third-party icons, reproduced without modification: the "healthy_liver" icon by Jan-Clusmann (https://www.linkedin.com/in/jan-clusmann/ is licensed under CC0 https://creativecommons.org/publicdomain/zero/1.0/) and the "mucous-neck-cell" icon by Servier (https://smart.servier.com/ is licensed under CC-BY 3.0 Unported https://creativecommons.org/licenses/by/3.0/).

## 7 Therapeutic strategies for hereditary diseases based on gut microbiota modulation

### 7.1 Dietary and nutritional interventions

The core concept of “precision nutrition” is to tailor dietary regimens according to individual genotypes and baseline microbiota profiles, so as to achieve optimal modulation of gut microbiota. Studies have demonstrated that dietary patterns act as critical regulators of the composition and function of gut microbiota. Distinct diets such as the Mediterranean diet and Western diet exert markedly different effects on microbial diversity and metabolites, including SCFAs and TMAO (182). The Mediterranean diet, rich in dietary fiber and plant protein, facilitates the proliferation of anti-inflammatory butyrate-producing bacteria and enhances microbial diversity, whereas the Western diet is linked to the overgrowth of pathogenic bacteria and gut dysbiosis (183). Collectively, precision dietary strategies based on host genetic background and baseline microbial characteristics represent a key direction for individualized health management in the future.

### 7.2 Precise application of prebiotics and probiotics

Next-generation probiotics (NGPs) shift from conventional strains to novel ones with well-defined functions, such as *Bacteroides ovatus*, *Akkermansia*, and *Faecalibacterium prausnitzii*, targeting specific gut microbiota defects (184). Dietary components (e.g., prebiotic fibers, polyphenols) enhance the colonization of these beneficial bacteria via pH, adhesion, and pathogen exclusion (185). This transition to functionally clarified NGPs offers new tools for targeted intervention against dysbiosis-associated diseases.

The design of synbiotics (probiotics + prebiotics) has become more rationalized to ensure colonization and function. A novel prebiotics-oriented screening strategy was developed; for example, *Lactobacillus* rhamnosus from young gut synergizes with xylo-oligosaccharides to reduce cholesterol *in vitro (*186). Such rational design highlights synergy, and its efficacy needs clinical trials considering host genetic backgrounds. For obesity, a low-calorie high-fiber diet (as prebiotics) combined with probiotic supplements (e.g., yogurt) improves body composition, decreases leptin, and increases *Lactobacillus/Bifidobacterium* abundance (187). Similarly, dietary patterns influence disease susceptibility via gut microbiota in children with atopic dermatitis, providing a basis for more effective probiotic regimens (188). Therefore, precise application of synbiotics based on host genetics and disease characteristics is critical to improve clinical efficacy (**Figure 9**).

**Figure 9:**
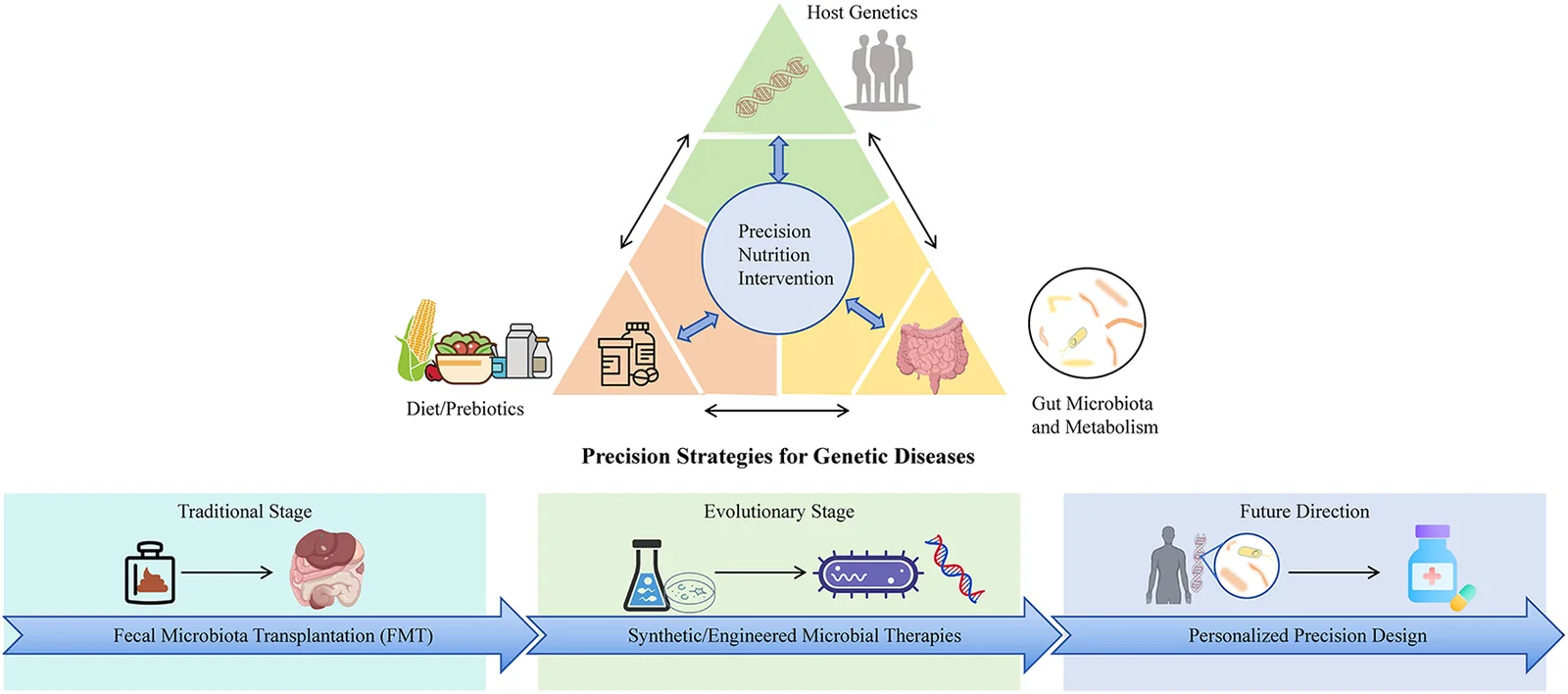
Therapeutic strategies for hereditary diseases based on gut microbiota modulation. Interplay among gene-diet-microbiota and precision intervention strategies for hereditary diseases. Diet serves as a key environmental regulator shaping the gut microbiome, and its biological effects are modulated by host genetic background. Integration of multi-omics datasets, including host genome, metagenome and metabolome, enables the construction of predictive models and facilitates the transition from general dietary recommendations to personalized nutrition regimens. For hereditary diseases, prebiotic supplementation can directionally increase beneficial microbial metabolites such as butyrate to compensate for microbial metabolic defects. Microbial therapeutic strategies have evolved from conventional FMT to well-defined synthetic/engineered living biotherapeutic products (LBPs), and further progress toward host-genetics-guided personalized precision design. Strain screening and engineering based on host genotypes such as HLA are performed to improve therapeutic efficacy and safety. This schematic incorporates the following third-party icons, reproduced without modification: the "DNA_monochrome" icon by DBCLS https://togotv.dbcls.jp/en/pics.html is licensed under CC-BY 4.0 Unported https://creativecommons.org/licenses/by/4.0/.

### 7.3 Microbial therapeutics: from FMT to precision engineered bacteria

FMT, a therapy for direct regulation of gut microecology, has achieved excellent efficacy in recurrent *Clostridioides difficile* infection. Its potential applications in genetic diseases are being extensively explored (189). For instance, FMT has the potential to induce remission in IBD, yet its efficacy varies markedly among individuals, highlighting the importance of microbiota matching between donors and recipients (190). Studies have indicated that the success of FMT relies not only on the quality of donor microbiota, but also closely correlates with the recipient’s genetic background, baseline gut microbiota composition and immune status (189). Such individual variations suggest that future microbial therapeutics should move beyond the one-size-fits-all model toward precision treatment. Next-generation microbial therapeutics are evolving toward the application of well-defined synthetic microbiota or single engineered strains (191). For example, a Phase I/IIa clinical trial demonstrated that oral administration of an engineered *E. coli Nissle* 1917 strain dose-dependently reduces blood phenylalanine levels in PKU patients, providing evidence for the clinical translation of live biotherapeutic products (192). The development of these advanced therapeutics urgently requires personalized design combined with host genetic information. For example, selecting strains that rarely trigger immune rejection or effectively modulate local immune responses according to patients’ immune genotypes such as human leukocyte antigen (HLA) haplotypes is essential for improving efficacy and safety (193). Complex interactions between host genetics and gut microbiota jointly determine the susceptibility to various diseases including autoimmune diseases of the central nervous system, which provides a theoretical basis for designing personalized microbial therapeutics based on host genotypes (**Figure 9**) (194).

### 7.4 Core barriers to clinical translation

Microbiota-targeted interventions for hereditary diseases currently face a structural evidence crisis: animal model data predominate absolutely, whereas large-scale human randomized controlled trials (RCTs) remain virtually nonexistent. Fundamental differences between mice and humans in cecal fermentation, immune development, and microbiota composition result in substantial attenuation or complete loss of efficacy for many strategies effective in rodents when translated to human trials (195). For microbiota interventions targeting Down syndrome, cystic fibrosis, phenylketonuria, and related diseases, no multicenter, randomized, double-blind, placebo-controlled trials with a sample size exceeding 100 participants and follow-up duration greater than 12 months have been conducted to date. The available evidence consists predominantly of open-label trials with fewer than 50 participants, rendering it impossible to exclude placebo effects and confounding factors. In the absence of high-quality RCTs, microbiota modulation should be regarded solely as an adjunctive strategy (196).

Three unresolved technical bottlenecks underlie this translational gap. First, the strain-specificity crisis: functional gene divergence among different strains of the same species (e.g., *Lactobacillus rhamnosus*) can reach 30%, resulting in markedly distinct SCFA production, adherence capacity, and immunomodulatory effects. Current genetic disease studies predominantly employ generic commercial strains without precise matching based on patient genotype–microbiota deficiency phenotypes, leading to irreproducible therapeutic outcomes (197). Second, the microbiota engraftment bottleneck: the colonization efficiency of FMT and defined consortia is constrained by multiple host factors, including FUT2 glycosylation status, HLA–mediated immune tolerance thresholds, and baseline microbiota diversity. In patients with hereditary diseases, engraftment efficiency is further compromised by hyperviscous intestinal mucus (CF), immune deviation (DS), or bioenergetic crisis (mitochondrial diseases), and predictive models for assessing colonization probability in specific donor–recipient pairings are currently lacking (198). Third, the safety blind spot for engineered bacteria: horizontal gene transfer (HGT) of recombinant metabolic genes may occur via conjugation or transduction into intestinal pathogens or environmental microbial reservoirs; prolonged colonization may induce antibody responses against foreign proteins and secondary dysbiosis; and metabolic bypass pathways may disrupt the homeostasis of essential amino acids such as tyrosine and tryptophan, with particularly elevated risks in pediatric and multi-system–affected patients (199). Existing Phase I/IIa trials have not conducted long-term monitoring of fecal gene drift, and regulatory approval pathways for LBPs together with disease-specific guidelines for genetic diseases remain incomplete at both the U.S. Food and Drug Administration (FDA) and European Medicines Agency (EMA) (200).

Future efforts should prioritize large-scale stratified RCTs targeting specific molecular subtypes of hereditary diseases, establishing a precision framework integrating host genotype, baseline microbiota, and intervention strategy as a unified triad, rather than simply replicating existing FMT or probiotic paradigms.

## 8 Conclusion

As a dynamic bridge linking host genetics and phenotypes, gut microbiota has evolved from a mere commensal community into a crucial mediator in the onset and progression of hereditary diseases. From monogenic diseases to complex polygenic diseases, gut dysbiosis can either amplify the pathogenic effects of genetic mutations or mitigate such impacts via metabolic compensation. This bidirectional regulatory mechanism reveals the limitations of the traditional genetic determinism theory. Host genes shape microbial ecology by regulating immune surveillance, intestinal barrier integrity and metabolic signaling networks. In turn, microbial metabolites such as SCFAs, bile acids and tryptophan derivatives modulate host gene expression via epigenetic modifications and signaling pathways, thereby forming an elaborate bidirectional interaction network. This finding suggests that phenotypic heterogeneity of hereditary diseases may partially stem from individual variations in microbiota composition, rather than being solely determined by genetic mutations.

In terms of intervention strategies, microbiota-modulating approaches including probiotics, fecal microbiota transplantation and engineered bacteria have shown therapeutic potential in certain hereditary diseases, yet clinical translation remains a major bottleneck. The current evidence relies heavily on animal models, with substantial deficits in large-scale, multicenter human RCT data. In the absence of high-quality RCT evidence, microbiota modulation should be positioned as an adjunctive strategy rather than a standalone therapeutic intervention. Conflicting findings across studies, such as inconsistent efficacy of fecal microbiota transplantation for IBD, indicate that the outcomes of microbiota intervention are highly dependent on host genetic background, baseline microbiota profile and disease stage. Accordingly, the one-size-fits-all intervention model should be abandoned in favor of precise regulation tailored to individual genotypes and microbial characteristics.

## References

[1] Farzand A Rohin MAK Awan SJ Sharif Z Yaseen A Ahmad AMR . A review of syndromic forms of obesity: Genetic etiology, clinical features, and molecular diagnosis. Curr Issues Mol Biol. (2025) 47:718. doi: 10.3390/cimb47090718 41020840 PMC12468879

[2] Sasahara Y Uchida T Suzuki T Abukawa D . Primary immunodeficiencies associated with early-onset inflammatory bowel disease in southeast and east asia. Front Immunol. (2021) 12:786538. doi: 10.3389/fimmu.2021.786538 35095863 PMC8792847

[3] Kirby TO Shi X Walters D Roullet JB Gibson KM . Intestinal dysbiosis as a component of pathophysiology in succinic semialdehyde dehydrogenase deficiency (ssadhd). Mol Genet Metab. (2022) 135:42–6. doi: 10.1016/j.ymgme.2021.11.355 34896003

[4] Lefter R Ciobica A Timofte D Stanciu C Trifan A . A descriptive review on the prevalence of gastrointestinal disturbances and their multiple associations in autism spectrum disorder. Med (Kaunas). (2019) 56:11. doi: 10.3390/medicina56010011 31892195 PMC7023358

[5] Wan Y Su Q Ng SC . New insights on gut microbiome and autism. Trends Mol Med. (2024) 30:1100–2. doi: 10.1016/j.molmed.2024.06.010 38987054

[6] Das S Patel M Khandelwal S Rawat R Shukla S Kumari AP . From mutations to microbes: Investigating the impact of the gut microbiome on repeat expansion disorders. J Neurochem. (2025) 169:e70278. doi: 10.1111/jnc.70278 41194479

[7] Syafarina I Mazaya M Indrawati A Akbar SZ Sukowati C Sadikin R . Skin microbial composition and genetic mutation analysis in precision medicine for epidermolysis bullosa. Curr Drug Targets. (2024) 25:404–15. doi: 10.2174/0113894501290512240327091531 38566380

[8] Manolis AA Manolis TA Melita H Manolis AS . Gut microbiota and cardiovascular disease: Symbiosis versus dysbiosis. Curr Med Chem. (2022) 29:4050–77. doi: 10.2174/0929867328666211213112949 34961453

[9] Li J Yang X Zhou X Cai J . The role and mechanism of intestinal flora in blood pressure regulation and hypertension development. Antioxid Redox Signal. (2021) 34:811–30. doi: 10.1089/ars.2020.8104 32316741

[10] Blanco C . The influence of the gut microbiome on obesity. J Am Assoc Nurse Pract. (2020) 32:504–10. doi: 10.1097/jxx.0000000000000480 32658171

[11] Fujisaka S Watanabe Y Tobe K . The gut microbiome: A core regulator of metabolism. J Endocrinol. (2023) 256:e220111. doi: 10.1530/joe-22-0111 36458804 PMC9874984

[12] Dhurjad P Dhavaliker C Gupta K Sonti R . Exploring Drug Metabolism by the Gut Microbiota: Modes of Metabolism and Experimental Approaches. Drug Metab Dispos. (2022) 50:224–34. doi: 10.1124/dmd.121.000669 34969660

[13] Akalin H Sahin IO Paskal SA Tan B Yalcinkaya E Demir M . Evaluation of chromosomal abnormalities in the postnatal cohort: A single-center study on 14,242 patients. J Clin Lab Anal. (2024) 38:e24997. doi: 10.1002/jcla.24997 38115218 PMC10829689

[14] Ren S Wang X Qin J Mu Q Ye S Zhang Y . Altered gut microbiota correlates with cognitive impairment in chinese children with down's syndrome. Eur Child Adolesc Psychiatry. (2022) 31:189–202. doi: 10.1007/s00787-021-01799-2 33999314 PMC8816804

[15] Rosas HD Morgan XC Tao Y Lai F Mercaldo ND . Gut dysbiosis in down syndrome: A potentially unexplored culprit for early alzheimer's disease. Alzheimers Dement. (2025) 21:e70330. doi: 10.1002/alz.70330 40491237 PMC12149442

[16] Hou X Wu N Ren S Wang X Mu Q Zhang Y . Profiling blautia at high taxonomic resolution reveals correlations with cognitive dysfunction in chinese children with down syndrome. Front Cell Infect Microbiol. (2023) 13:1109889. doi: 10.3389/fcimb.2023.1109889 36844413 PMC9950735

[17] Cai S Lin J Li Z Liu S Feng Z Zhang Y . Alterations in intestinal microbiota and metabolites in individuals with down syndrome and their correlation with inflammation and behavior disorders in mice. Front Microbiol. (2023) 14:1016872. doi: 10.3389/fmicb.2023.1016872 36910172 PMC9998045

[18] Zhou Y Zhang F Mao L Feng T Wang K Xu M . Bifico relieves irritable bowel syndrome by regulating gut microbiota dysbiosis and inflammatory cytokines. Eur J Nutr. (2023) 62:139–55. doi: 10.1007/s00394-022-02958-0 35918555 PMC9899748

[19] Zhang D Zhu C Fang Z Zhang H Yang J Tao K . Remodeling gut microbiota by clostridium butyricum (c.Butyricum) attenuates intestinal injury in burned mice. Burns. (2020) 46:1373–80. doi: 10.1016/j.burns.2020.01.007 32014349

[20] Shen SH Zhong TY Peng C Fang J Lv B . Structural modulation of gut microbiota during alleviation of non-alcoholic fatty liver disease with gynostemma pentaphyllum in rats. BMC Complement Med Ther. (2020) 20:34. doi: 10.1186/s12906-020-2835-7 32024509 PMC7076883

[21] Edmondson C Course CW Doull I . Cystic fibrosis transmembrane conductance regulator modulators for cystic fibrosis: A new dawn? Arch Dis Child. (2021) 106:941–5. doi: 10.1136/archdischild-2020-320680 33785533

[22] Meeker SM Mears KS Sangwan N Brittnacher MJ Weiss EJ Treuting PM . Cftr dysregulation drives active selection of the gut microbiome. PLoS Pathog. (2020) 16:e1008251. doi: 10.1371/journal.ppat.1008251 31961914 PMC6994172

[23] Rigauts C Aizawa J Taylor SL Rogers GB Govaerts M Cos P . R othia mucilaginosa is an anti-inflammatory bacterium in the respiratory tract of patients with chronic lung disease. Eur Respir J. (2022) 59:2101293. doi: 10.1183/13993003.01293-2021 34588194 PMC9068977

[24] Tam RY van Dorst JM McKay I Coffey M Ooi CY . Intestinal inflammation and alterations in the gut microbiota in cystic fibrosis: A review of the current evidence, pathophysiology and future directions. J Clin Med. (2022) 11:649. doi: 10.3390/jcm11030649 35160099 PMC8836727

[25] Frey DL Boutin S Dittrich SA Graeber SY Stahl M Wege S . Relationship between airway dysbiosis, inflammation and lung function in adults with cystic fibrosis. J Cyst Fibros. (2021) 20:754–60. doi: 10.1016/j.jcf.2020.12.022 33431308

[26] Linnane B Walsh AM Walsh CJ Crispie F O'Sullivan O Cotter PD . The lung microbiome in young children with cystic fibrosis: A prospective cohort study. Microorganisms. (2021) 9:492. doi: 10.3390/microorganisms9030492 33652802 PMC7996874

[27] Ray KJ Santee C McCauley K Panzer AR Lynch SV . Gut bifidobacteria enrichment following oral lactobacillus-supplementation is associated with clinical improvements in children with cystic fibrosis. BMC Pulm Med. (2022) 22:287. doi: 10.1186/s12890-022-02078-9 35902830 PMC9330662

[28] Woods DF Flynn S Caparrós-Martín JA Stick SM Reen FJ O'Gara F . Systems biology and bile acid signalling in microbiome-host interactions in the cystic fibrosis lung. Antibiotics (Basel). (2021) 10:766. doi: 10.3390/antibiotics10070766 34202495 PMC8300688

[29] Flynn S Reen FJ Caparrós-Martín JA Woods DF Peplies J Ranganathan SC . Bile acid signal molecules associate temporally with respiratory inflammation and microbiome signatures in clinically stable cystic fibrosis patients. Microorganisms. (2020) 8:1741. doi: 10.3390/microorganisms8111741 33172004 PMC7694639

[30] Biondi A Basile F Vacante M . Familial adenomatous polyposis and changes in the gut microbiota: New insights into colorectal cancer carcinogenesis. World J Gastrointest Oncol. (2021) 13:495–508. doi: 10.4251/wjgo.v13.i6.495 34163569 PMC8204352

[31] Kim JH Kim YJ Oh GM Jung W Park SJ . How is gut microbiome of patients with familial adenomatous polyposis different from healthy people? Med (Baltimore). (2022) 101:e32194. doi: 10.1097/md.0000000000032194 36626451 PMC9750518

[32] Liang S Mao Y Liao M Xu Y Chen Y Huang X . Gut microbiome associated with apc gene mutation in patients with intestinal adenomatous polyps. Int J Biol Sci. (2020) 16:135–46. doi: 10.7150/ijbs.37399 31892851 PMC6930378

[33] Aceto GM Catalano T Curia MC . Molecular aspects of colorectal adenomas: The interplay among microenvironment, oxidative stress, and predisposition. BioMed Res Int. (2020) 2020:1726309. doi: 10.1155/2020/1726309 32258104 PMC7102468

[34] Xu C Gu L Hu L Jiang C Li Q Sun L . Fads1-arachidonic acid axis enhances arachidonic acid metabolism by altering intestinal microecology in colorectal cancer. Nat Commun. (2023) 14:2042. doi: 10.1038/s41467-023-37590-x 37041160 PMC10090135

[35] Lavoie S Chun E Bae S Brennan CA Gallini Comeau CA Lang JK . Expression of free fatty acid receptor 2 by dendritic cells prevents their expression of interleukin 27 and is required for maintenance of mucosal barrier and immune response against colorectal tumors in mice. Gastroenterology. (2020) 158:1359–1372.e1359. doi: 10.1053/j.gastro.2019.12.027 31917258 PMC7291292

[36] Li X Qian J Liu Q Guo M Zhang H Li H . Yogurt prevents colorectal tumorigenesis in apc(min/+) mice. Mol Nutr Food Res. (2024) 68:e2300737. doi: 10.1002/mnfr.202300737 38700077

[37] Zhou L Zheng L Xu B . Clinical efficacy of metformin in familial adenomatous polyposis and the effect of intestinal flora. Orphanet J Rare Dis. (2024) 19:88. doi: 10.1186/s13023-024-03064-6 38403687 PMC10895836

[38] Cacicedo ML Weinl-Tenbruck C Frank D . Phenylalanine hydroxylase mrna rescues the phenylketonuria phenotype in mice. Front Bioeng Biotechnol. (2022) 10:993298. doi: 10.3389/fbioe.2022.993298 36277393 PMC9585315

[39] Montanari C Ceccarani C Corsello A . Glycomacropeptide safety and its effect on gut microbiota in patients with phenylketonuria: A pilot study. Nutrients. (2022) 14:1883. doi: 10.3390/nu14091883 35565850 PMC9104775

[40] Mancilla VJ Mann AE Zhang Y . The adult phenylketonuria (pku) gut microbiome. Microorganisms. (2021) 9:530. doi: 10.3390/microorganisms9030530 33806544 PMC8001843

[41] Jiang Y Sun B Qian F . Expression of phenylalanine ammonia lyase as an intracellularly free and extracellularly cell surface-immobilized enzyme on a gut microbe as a live biotherapeutic for phenylketonuria. Sci China Life Sci. (2023) 66:127–36. doi: 10.1007/s11427-021-2137-3 35907113 PMC9362719

[42] Liang J Yang S Liu Y . Characterization and stability assessment of polyphenols bound to lycium barbarum polysaccharide: Insights from gastrointestinal digestion and colon fermentation. Food Res Int. (2024) 179:114036. doi: 10.1016/j.foodres.2024.114036 38342549

[43] Salekeen R Siam MHB Sharif DI . In silico insights into potential gut microbial modulation of nad+ metabolism and longevity. J Biochem Mol Toxicol. (2021) 35:e22925. doi: 10.1002/jbt.22925 34580953

[44] Povea-Cabello S Villanueva-Paz M Suárez-Rivero JM . Advances in mt-trna mutation-caused mitochondrial disease modeling: Patients' brain in a dish. Front Genet. (2020) 11:610764. doi: 10.3389/fgene.2020.610764 33510772 PMC7835939

[45] Banerjee S Mondal R Deb S . Integrated molecular and clinical profiling of primary mitochondrial oxidative phosphorylation disorders in an indian cohort: Insights from genetics, neuroimaging, and machine learning. Mitochondrion. (2026) 89:102150. doi: 10.1016/j.mito.2026.102150 41850596

[46] Rahman A Faiz M Khalid M . Gut instinct: Microbiome as a modifiable target in the management of neurologic symptoms in myoclonic epilepsy with ragged-red fibers (merrf). Ann Med Surg (Lond). (2025) 87:6904–5. doi: 10.1097/ms9.0000000000003777 41181495 PMC12577839

[47] Nagy-Grócz G Spekker E Vécsei L . Kynurenines, neuronal excitotoxicity, and mitochondrial oxidative stress: Role of the intestinal flora. Int J Mol Sci. (2024) 25:1698. doi: 10.3390/ijms25031698 38338981 PMC10855176

[48] Mohammed V Muthuramamoorthy M Arasu MV Arockiaraj J . Gut microbiome and mitochondrial crosstalk in schizophrenia, a mental disability: Emerging mechanisms and therapeutic targets. Neurosci Biobehav Rev. (2025) 178:106371. doi: 10.1016/j.neubiorev.2025.106371 40930337

[49] Guo P Zeng M Liu M Zhang Y Jia J Zhang Z . Isolation of calenduloside e from achyranthes bidentata blume and its effects on lps/d-galn-induced acute liver injury in mice by regulating the ampk-sirt3 signaling pathway. Phytomedicine. (2024) 125:155353. doi: 10.1016/j.phymed.2024.155353 38241918

[50] Samant SA Hyoju SK Alverdy JC Gupta MP . The multifaceted role of sirtuins in inflammatory bowel diseases. Am J Physiol Gastrointest Liver Physiol. (2025) 329:G58–g68. doi: 10.1152/ajpgi.00311.2024 40298096 PMC12199837

[51] Nayar S Morrison JK Giri M Gettler K Chuang LS Walker LA . A myeloid-stromal niche and gp130 rescue in nod2-driven crohn's disease. Nature. (2021) 593:275–81. doi: 10.1038/s41586-021-03484-5 33789339 PMC8284870

[52] Pisani A Rausch P Bang C Ellul S Tabone T Marantidis Cordina C . Dysbiosis in the gut microbiota in patients with inflammatory bowel disease during remission. Microbiol Spectr. (2022) 10:e0061622. doi: 10.1128/spectrum.00616-22 35532243 PMC9241752

[53] Vestergaard MV Allin KH Eriksen C Zakerska-Banaszak O Arasaradnam RP Alam MT . Gut microbiota signatures in inflammatory bowel disease. United Eur Gastroenterol J. (2024) 12:22–33. doi: 10.1002/ueg2.12485 38041519 PMC10859715

[54] Akiyama S Nishijima S Kojima Y Kimura M Ohsugi M Ueki K . Multi-biome analysis identifies distinct gut microbial signatures and their crosstalk in ulcerative colitis and crohn's disease. Nat Commun. (2024) 15:10291. doi: 10.1038/s41467-024-54797-8 39604394 PMC11603027

[55] Kaur S Thakur R . Gut microbiota and inflammatory bowel disease: Mechanisms, clinical implications, and future directions. Mol Biol Rep. (2025) 53:47. doi: 10.1007/s11033-025-11195-7 41191152

[56] Serrano-Gómez G Yañez F Soler Z Pons-Tarin M Mayorga L Herrera-deGuise C . Microbiome multi-omics analysis reveals novel biomarkers and mechanisms linked with cd etiopathology. biomark Res. (2025) 13:85. doi: 10.1186/s40364-025-00802-1 40524271 PMC12172205

[57] Saiz-Gonzalo G Hanrahan N Rossini V Singh R Ahern M Kelleher M . Regulation of ceacam family members by ibd-associated triggers in intestinal epithelial cells, their correlation to inflammation and relevance to ibd pathogenesis. Front Immunol. (2021) 12:655960. doi: 10.3389/fimmu.2021.655960 34394073 PMC8358819

[58] Kinugasa T Tsunoda T Mizoguchi E Okada T Sudo T Kawahara A . Chitinase 3-like 1, carcinoembryonic antigen-related cell adhesion molecule 6, and ectopic claudin-2 in the carcinogenic processes of ulcerative colitis. Anticancer Res. (2022) 42:4119–27. doi: 10.21873/anticanres.15910 35896264

[59] Nag D Farr D Raychaudhuri S Withey JH . An adult zebrafish model for adherent-invasive escherichia coli indicates protection from aiec infection by probiotic e. coli nissle. iScience. (2022) 25:104572. doi: 10.1016/j.isci.2022.104572 35769878 PMC9234234

[60] Paroni M Leccese G Ranzani V Moschetti G Chiara M Perillo F . An intestinal th17 subset is associated with inflammation in crohn's disease and activated by adherent-invasive escherichia coli. J Crohns Colitis. (2023) 17:1988–2001. doi: 10.1093/ecco-jcc/jjad119 PMC10798865 37462681

[61] Roussin L Prince N Perez-Pardo P Kraneveld AD Rabot S Naudon L . Role of the gut microbiota in the pathophysiology of autism spectrum disorder: Clinical and preclinical evidence. Microorganisms. (2020) 8:1369. doi: 10.3390/microorganisms8091369 32906656 PMC7563175

[62] Hua X Zhu J Yang T Guo M Li Q Chen J . The gut microbiota and associated metabolites are altered in sleep disorder of children with autism spectrum disorders. Front Psychiatry. (2020) 11:855. doi: 10.3389/fpsyt.2020.00855 32982808 PMC7493623

[63] Li Y Hu W Lin B Ma T Zhang Z Hu W . Omic characterizing and targeting gut dysbiosis in children with autism spectrum disorder: Symptom alleviation through combined probiotic and medium-carbohydrate diet intervention - a pilot study. Gut Microbes. (2024) 16:2434675. doi: 10.1080/19490976.2024.2434675 39632378 PMC11622613

[64] Kandeel WA Meguid NA Bjørklund G Eid EM Farid M Mohamed SK . Impact of clostridium bacteria in children with autism spectrum disorder and their anthropometric measurements. J Mol Neurosci. (2020) 70:897–907. doi: 10.1007/s12031-020-01482-2 32130666

[65] Chen Z Shi K Liu X Dai Y Liu Y Zhang L . Gut microbial profile is associated with the severity of social impairment and iq performance in children with autism spectrum disorder. Front Psychiatry. (2021) 12:789864. doi: 10.3389/fpsyt.2021.789864 34975585 PMC8718873

[66] Jung Y Lee T Oh HS Hyun Y Song S Chun J . Gut microbial and clinical characteristics of individuals with autism spectrum disorder differ depending on the ecological structure of the gut microbiome. Psychiatry Res. (2024) 335:115775. doi: 10.1016/j.psychres.2024.115775 38503005

[67] Dargenio VN Dargenio C Castellaneta S De Giacomo A Laguardia M Schettini F . Intestinal barrier dysfunction and microbiota-gut-brain axis: Possible implications in the pathogenesis and treatment of autism spectrum disorder. Nutrients. (2023) 15:1620. doi: 10.3390/nu15071620 37049461 PMC10096948

[68] Sulas F Favero G Bonini SA Lonati C Pinto D Memo M . Melatonin improves intestinal barrier impairment in a mouse model of autism spectrum disorder. Biol (Basel). (2025) 14:1594. doi: 10.3390/biology14111594 41300382 PMC12650372

[69] Longo B Nunes RKS Cazarin CA Silva T Sievers J Nilz A . Valproic acid-induced autistic-like behavior is accompanied by intestinal damage driving changes in gut permeability in a sex-dependent way in rats. J Neurochem. (2025) 169:e70316. doi: 10.1111/jnc.70316 41392905 PMC12703684

[70] Rowshan N Anjomshoa M Farahzad A Bijad E Amini-Khoei H . Gut-brain barrier dysfunction bridge autistic-like behavior in mouse model of maternal separation stress: A behavioral, histopathological, and molecular study. Int J Dev Neurosci. (2024) 84:314–27. doi: 10.1002/jdn.10329 38584149

[71] Hu C Li H Cui J Li Y Zhang F Li H . Integrative analysis identifies il-6/jun/mmp-9 pathway destroyed blood-brain-barrier in autism mice via machine learning and bioinformatic analysis. Transl Psychiatry. (2025) 15:239. doi: 10.1038/s41398-025-03452-x 40645949 PMC12254206

[72] Loos RJF Yeo GSH . The genetics of obesity: From discovery to biology. Nat Rev Genet. (2022) 23:120–33. doi: 10.1038/s41576-021-00414-z 34556834 PMC8459824

[73] Amabebe E Robert FO Agbalalah T Orubu ESF . Microbial dysbiosis-induced obesity: Role of gut microbiota in homoeostasis of energy metabolism. Br J Nutr. (2020) 123:1127–37. doi: 10.1017/s0007114520000380 32008579

[74] Moughaizel M Dagher E Jablaoui A Thorin C Rhimi M Desfontis JC . Long-term high-fructose high-fat diet feeding elicits insulin resistance, exacerbates dyslipidemia and induces gut microbiota dysbiosis in whhl rabbits. PLoS One. (2022) 17:e0264215. doi: 10.1371/journal.pone.0264215 35196347 PMC8865649

[75] Gupta R Saini P Srivastava V Ittishree Paramasivam K Singh R . Microbiota-derived metabolites as nutritional signals in insulin resistance and obesity. Clin Nutr ESPEN. (2026) 75:103279. doi: 10.1016/j.clnesp.2026.103279 41974372

[76] Riggen-Bueno V Del Toro-Arreola S Baltazar-Díaz TA Vega-Magaña AN Peña-Rodríguez M Castaño-Jiménez PA . Intestinal dysbiosis in subjects with obesity from western mexico and its association with a proinflammatory profile and disturbances of folate (b9) and carbohydrate metabolism. Metabolites. (2024) 14:121. doi: 10.3390/metabo14020121 38393013 PMC10890169

[77] Lupu VV Nedelcu AH Borka-Balas R Anton CR Tarnita I Azoicai A . The gut microbiota: An essential component in understanding pediatric obesity: A narrative review. Nutrients. (2026) 18:952. doi: 10.3390/nu18060952 41901128 PMC13029728

[78] Takeuchi T Kubota T Nakanishi Y Tsugawa H Suda W Kwon AT . Gut microbial carbohydrate metabolism contributes to insulin resistance. Nature. (2023) 621:389–95. doi: 10.1038/s41586-023-06466-x 37648852 PMC10499599

[79] Choi BS Daniel N Houde VP Ouellette A Marcotte B Varin TV . Feeding diversified protein sources exacerbates hepatic insulin resistance via increased gut microbial branched-chain fatty acids and mtorc1 signaling in obese mice. Nat Commun. (2021) 12:3377. doi: 10.1038/s41467-021-23782-w 34099716 PMC8184893

[80] LiY ZhangY LiuT TuoY ZhangY YouH . Alleviation of high-fat diet-induced lipid metabolism disorders: Role of quinoa peptides in reducing high-activity bsh-producing gut microbiota abundance and modulating ba-fxr/tgr5 signaling. J Adv Res. (2026) 17:S2090–1232. doi: 10.1016/j.jare.2026.02.04041713730

[81] Liu Q Tian H Kang Y Tian Y Li L Kang X . Probiotics alleviate autoimmune hepatitis in mice through modulation of gut microbiota and intestinal permeability. J Nutr Biochem. (2021) 98:108863. doi: 10.1016/j.jnutbio.2021.108863 34517094

[82] Majumder S Pushpakumar SB Almarshood H Ouseph R Gondim DD Jala VR . Toll-like receptor 4 mutation mitigates gut microbiota-mediated hypertensive kidney injury. Pharmacol Res. (2024) 206:107303. doi: 10.1016/j.phrs.2024.107303 39002869 PMC11287947

[83] Castagnoli R Pala F Bosticardo M Licari A Delmonte OM Villa A . Gut microbiota-host interactions in inborn errors of immunity. Int J Mol Sci. (2021) 22:1416. doi: 10.3390/ijms22031416 33572538 PMC7866830

[84] Soejima M Koda Y . Detection of the weak-secretor rs1047781 (385a>t) single nucleotide polymorphism using an unlabeled probe high-resolution melting-based method. Electrophoresis. (2021) 42:1362–5. doi: 10.1002/elps.202000386 33835481

[85] Cheng S Hu J Wu X Pan JA Jiao N Li Y . Altered gut microbiome in fut2 loss-of-function mutants in support of personalized medicine for inflammatory bowel diseases. J Genet Genomics. (2021) 48:771–80. doi: 10.1016/j.jgg.2021.08.003 34419617

[86] Battat R Qatomah A Kopylov U Wyse J Cohen A Afif W . Fucosyltransferase 2 mutations are associated with a favorable clinical course in crohn's disease. J Clin Gastroenterol. (2022) 56:e166–70. doi: 10.1097/mcg.0000000000001626 34739405

[87] Dekkers KF Pertiwi K Baldanzi G Lundmark P Hammar U Moksnes MR . Genome-wide association analyses highlight the role of the intestinal molecular environment in human gut microbiota variation. Nat Genet. (2026) 58:540–9. doi: 10.1038/s41588-026-02512-2 41688638 PMC12987725

[88] Liu X Tang S Zhong H Tong X Jie Z Ding Q . A genome-wide association study for gut metagenome in chinese adults illuminates complex diseases. Cell Discov. (2021) 7:9. doi: 10.1038/s41421-020-00239-w 33563976 PMC7873036

[89] Gacesa R Kurilshikov A Vich Vila A Sinha T Klaassen MAY Bolte LA . Environmental factors shaping the gut microbiome in a dutch population. Nature. (2022) 604:732–9. doi: 10.1038/s41586-022-04567-7 35418674

[90] Ahmad F Saha P Singh V Wahid M Mandal RK Nath Mishra B . Diet as a modifiable factor in tumorigenesis: Focus on microbiome-derived bile acid metabolites and short-chain fatty acids. Food Chem. (2023) 410:135320. doi: 10.1016/j.foodchem.2022.135320 36610090

[91] de Vos WM Tilg H Van Hul M Cani PD . Gut microbiome and health: Mechanistic insights. Gut. (2022) 71:1020–32. doi: 10.1136/gutjnl-2021-326789 35105664 PMC8995832

[92] Zhang Y Xiao X Guo J Lei X Xiong W Wang S . Multidimensional regulatory mechanisms and translational potential of epigenetic networks in the rheumatoid arthritis disease course. Front Immunol. (2026) 17:1792863. doi: 10.3389/fimmu.2026.1792863 41929505 PMC13038445

[93] Kumar V Shukla R Gangani S Joseph R Jain S Yadav H . Epigenetics and the gut-brain axis: Insights into DNA methylation, aging, and alzheimer disease. J Pharmacol Exp Ther. (2026) 393:104299. doi: 10.1016/j.jpet.2026.104299 41886887

[94] Rathour D Shah S Khan S Singh PK Srivastava S Singh SB . Role of gut microbiota in depression: Understanding molecular pathways, recent research, and future direction. Behav Brain Res. (2023) 436:114081. doi: 10.1016/j.bbr.2022.114081 36037843

[95] Nunna Sai Venkata L Mishra AK Mohanta YK Rustagi S Bahuguna A Tomar A . The gut gambit: A review of how microbial imbalance fuels metabolic mayhem. Nutrients. (2026) 18:888. doi: 10.3390/nu18060888 41901063 PMC13029746

[96] Chervy M Sivignon A Dambrine F Buisson A Sauvanet P Godfraind C . Epigenetic master regulators hdac1 and hdac5 control pathobiont enterobacteria colonization in ileal mucosa of crohn's disease patients. Gut Microbes. (2022) 14:2127444. doi: 10.1016/s0016-5085(22)61659-7 PMC9542275 36175163

[97] Ryan FJ Ahern AM Fitzgerald RS Laserna-Mendieta EJ Power EM Clooney AG . Colonic microbiota is associated with inflammation and host epigenomic alterations in inflammatory bowel disease. Nat Commun. (2020) 11:1512. doi: 10.1038/s41467-020-15342-5 32251296 PMC7089947

[98] Dey P . Targeting gut barrier dysfunction with phytotherapies: Effective strategy against chronic diseases. Pharmacol Res. (2020) 161:105135. doi: 10.1016/j.phrs.2020.105135 32814166

[99] Kinashi Y Hase K . Partners in leaky gut syndrome: Intestinal dysbiosis and autoimmunity. Front Immunol. (2021) 12:673708. doi: 10.3389/fimmu.2021.673708 33968085 PMC8100306

[100] Togao M Asakawa N Wagai G Ohta-Takada Y Otsuka J Ando M . Dual effects of indoxyl sulfate on modulation of human hepatic cyp3a activity, with individual differences. PLoS One. (2025) 20:e0328182. doi: 10.1371/journal.pone.0328182 40638708 PMC12244530

[101] Behrouzi A Ashrafian F Mazaheri H Lari A Nouri M Riazi Rad F . The importance of interaction between micrornas and gut microbiota in several pathways. Microb Pathog. (2020) 144:104200. doi: 10.1016/j.micpath.2020.104200 32289465

[102] Liu L Wang H Yu Y Zeng B Rao X Chen J . Microbial regulation of a lincrna-mirna-mrna network in the mouse hippocampus. Epigenomics. (2020) 12:1377–87. doi: 10.2217/epi-2019-0307 32878473

[103] Zhao K Hu J Jiang L Zhao R Wang D Yang Y . Polysaccharide from walnut green husk alleviates ochratoxin a-induced anorexia in chicks via m(6)a-mediated fxr regulation of orexigenic genes. Poult Sci. (2026) 105:106402. doi: 10.1016/j.psj.2026.106402 41518909 PMC12819036

[104] Meng K Bao Y Chen G Qu J Liang S An S . Metagenomics and transcriptomics analysis of aspartame's impact on gut microbiota and glioblastoma progression in a mouse model. Sci Rep. (2025) 15:23298. doi: 10.1038/s41598-025-06193-5 40604039 PMC12223035

[105] Wang C Bai J Chen X Song J Zhang Y Wang H . Gut microbiome-based strategies for host health and disease. Crit Rev Food Sci Nutr. (2024) 64:6834–49. doi: 10.1080/10408398.2023.2176464 36803092

[106] Deng W Ke H Wang S Li Z Li S Lv P . Metformin alleviates autistic-like behaviors elicited by high-fat diet consumption and modulates the crosstalk between serotonin and gut microbiota in mice. Behav Neurol. (2022) 2022:6711160. doi: 10.1155/2022/6711160 35222739 PMC8872653

[107] Liu D Gao X Huang X Fan Y Wang YE Zhang Y . Moderate altitude exposure impacts host fasting blood glucose and serum metabolome by regulation of the intestinal flora. Sci Total Environ. (2023) 905:167016. doi: 10.1016/j.scitotenv.2023.167016 37714338

[108] Chunduri A Reddy SDM Jahanavi M Reddy CN . Gut-brain axis, neurodegeneration and mental health: A personalized medicine perspective. Indian J Microbiol. (2022) 62:505–15. doi: 10.1007/s12088-022-01033-w 36458229 PMC9705676

[109] Tian Y Jin W Jin X Wang Y Wu R Yu R . Fecal microbiota transplantation promotes hair growth through gut microbiome and metabolic regulation. Life Sci. (2025) 379:123887. doi: 10.1016/j.lfs.2025.123887 40749821

[110] Khasanov R Boettcher M Wessel LM Schäfer KH Tapia-Laliena M . All roads lead to nf-κb: The nf-κb pathway as a major target for intestinal inflammatory disorders. Front Immunol. (2026) 17:1816653. doi: 10.3389/fimmu.2026.1816653 42148126 PMC13171789

[111] Peña-Cearra A Castelo J Lavín JL Gonzalez-Lopez M Pascual-Itoiz MA Fuertes M . Mitochondrial dysfunction-associated microbiota establishes a transmissible refractory response to anti-tnf therapy during ulcerative colitis. Gut Microbes. (2023) 15:2266626. doi: 10.1080/19490976.2023.2266626 37842919 PMC10586225

[112] Chen Y Zhou J Wang L . Role and mechanism of gut microbiota in human disease. Front Cell Infect Microbiol. (2021) 11:625913. doi: 10.3389/fcimb.2021.625913 33816335 PMC8010197

[113] Cao Q Shen M Li R Liu Y Zeng Z Zhou J . Elucidating the specific mechanisms of the gut-brain axis: The short-chain fatty acids-microglia pathway. J Neuroinflamm. (2025) 22:133. doi: 10.1186/s12974-025-03454-y 40400035 PMC12093714

[114] Tran SM Mohajeri MH . The role of gut bacterial metabolites in brain development, aging and disease. Nutrients. (2021) 13:732. doi: 10.3390/nu13030732 33669008 PMC7996516

[115] Beopoulos A Gea M Fasano A Iris F . Autonomic nervous system neuroanatomical alterations could provoke and maintain gastrointestinal dysbiosis in autism spectrum disorder (asd): A novel microbiome-host interaction mechanistic hypothesis. Nutrients. (2021) 14:65. doi: 10.3390/nu14010065 35010940 PMC8746684

[116] Chen Y Xu J Chen Y . Regulation of neurotransmitters by the gut microbiota and effects on cognition in neurological disorders. Nutrients. (2021) 13:2099. doi: 10.3390/nu13062099 34205336 PMC8234057

[117] Violi F Cammisotto V Bartimoccia S Pignatelli P Carnevale R Nocella C . Gut-derived low-grade endotoxaemia, atherothrombosis and cardiovascular disease. Nat Rev Cardiol. (2023) 20:24–37. doi: 10.1038/s41569-022-00737-2 35840742 PMC9284488

[118] Socała K Doboszewska U Szopa A Serefko A Włodarczyk M Zielińska A . The role of microbiota-gut-brain axis in neuropsychiatric and neurological disorders. Pharmacol Res. (2021) 172:105840. doi: 10.1016/j.phrs.2021.105840 34450312

[119] Miao ZY Chen SF Wu H Liu XY Shao HY . Analysis of genetic characteristics of 436 children with dysplasia and detailed analysis of rare karyotype. Open Life Sci. (2022) 17:416–25. doi: 10.1515/biol-2022-0046 35582623 PMC9055171

[120] Gensous N Bacalini MG Franceschi C Garagnani P . Down syndrome, accelerated aging and immunosenescence. Semin Immunopathol. (2020) 42:635–45. doi: 10.1007/s00281-020-00804-1 32705346 PMC7666319

[121] Gur M Zuckerman-Levin N Masarweh K Hanna M Laghi L Marazzato M . The effect of probiotic administration on metabolomics and glucose metabolism in cf patients. Pediatr Pulmonol. (2022) 57:2335–43. doi: 10.1002/ppul.26037 35676769 PMC9796051

[122] Chen G Li Y Wei S Wang X Kuang Z Guo W . Role of gut microbiota in thalassemia: A review of therapeutic prospects. Front Physiol. (2025) 16:1523448. doi: 10.3389/fphys.2025.1523448 40177354 PMC11962020

[123] Gupta CL Jaganathasamy N Madkaikar M . Microbiome in sickle cell disease: Pathophysiology and therapeutic insights. Br J Haematol. (2024) 205:1279–87. doi: 10.1111/bjh.19736 39206530

[124] Ghalwash AA El-Gohary RM El Amrousy D Morad LM Kassem SS Hegab II . The gut microbiota metabolite trimethylamine-n-oxide in children with β-thalassemia: Potential implication for iron-induced renal tubular dysfunction. Pediatr Res. (2025) 98:621–8. doi: 10.1038/s41390-024-03639-w 39448817 PMC12454125

[125] Brim H Taylor J Abbas M Vilmenay K Daremipouran M Varma S . The gut microbiome in sickle cell disease: Characterization and potential implications. PLoS One. (2021) 16:e0255956. doi: 10.1371/journal.pone.0255956 34432825 PMC8386827

[126] Agbalalah T Bur D Nwonu EJ Rowaiye AB . Gut microbiota: Potential therapeutic target for sickle cell disease pain and complications. Anemia. (2024) 2024:5431000. doi: 10.1155/2024/5431000 38533265 PMC10965282

[127] Nonejuie P Wilantho A McDonald D Htoo HH Chalerm J Tripathi A . Differential gut microbiota composition in β-thalassemia patients and its correlation with iron overload. Sci Rep. (2024) 14:23858. doi: 10.1038/s41598-024-75456-4 39394230 PMC11470119

[128] Goel A Gupta M Aggarwal R . Gut microbiota and liver disease. J Gastroenterol Hepatol. (2014) 29:1139–48. doi: 10.1111/jgh.12556 24547986

[129] Sriwichaiin S Thiennimitr P Thonusin C Sarichai P Buddhasiri S Kumfu S . Deferiprone has less benefits on gut microbiota and metabolites in high iron-diet induced iron overload thalassemic mice than in iron overload wild-type mice: A preclinical study. Life Sci. (2022) 307:120871. doi: 10.1016/j.lfs.2022.120871 35952729

[130] Del Cornò M Aureli A Varano B Conti L . Endotoxins and metabolic endotoxemia in obesity and associated noncommunicable diseases: A focus on sex differences. Biomolecules. (2026) 16:226. doi: 10.3390/biom16020226 41750295 PMC12938599

[131] Bolívar BE Brown-Suedel AN Rohrman BA Charendoff CI Yazdani V Belcher JD . Noncanonical roles of caspase-4 and caspase-5 in heme-driven il-1β release and cell death. J Immunol. (2021) 206:1878–89. doi: 10.4049/jimmunol.2000226 PMC8026643 33741688

[132] Santana SS Pitanga TN de Santana JM Zanette DL Vieira JJ Yahouédéhou S . Hydroxyurea scavenges free radicals and induces the expression of antioxidant genes in human cell cultures treated with hemin. Front Immunol. (2020) 11:1488. doi: 10.3389/fimmu.2020.01488 32765515 PMC7380266

[133] Visitchanakun P Saisorn W Wongphoom J Chatthanathon P Somboonna N Svasti S . Gut leakage enhances sepsis susceptibility in iron-overloaded β-thalassemia mice through macrophage hyperinflammatory responses. Am J Physiol Gastrointest Liver Physiol. (2020) 318:G966–g979. doi: 10.1152/ajpgi.00337.2019 32308038

[134] Yang L Slone J Zou W Queme LF Jankowski MP Yin F . Systemic delivery of aav-fdxr mitigates the phenotypes of mitochondrial disorders in fdxr mutant mice. Mol Ther Methods Clin Dev. (2020) 18:84–97. doi: 10.1016/j.omtm.2020.05.021 32995353 PMC7488755

[135] Zachos KA Gamboa JA Dewji AS Lee J Brijbassi S Andreazza AC . The interplay between mitochondria, the gut microbiome and metabolites and their therapeutic potential in primary mitochondrial disease. Front Pharmacol. (2024) 15:1428242. doi: 10.3389/fphar.2024.1428242 39119601 PMC11306032

[136] Lu Y Chen X Lai Y . The development and emerging trends in parkinson's disease from the perspective of mitochondrial dysfunction: A bibliometric analysis using citespace. J Neurol. (2025) 272:721. doi: 10.1007/s00415-025-13472-8 41125853

[137] Zhang H Zha X Zhang B Zheng Y Elsabagh M Wang H . Gut microbiota contributes to bisphenol a-induced maternal intestinal and placental apoptosis, oxidative stress, and fetal growth restriction in pregnant ewe model by regulating gut-placental axis. Microbiome. (2024) 12:28. doi: 10.1186/s40168-024-01749-5 38365714 PMC10874076

[138] Lobzhanidze G Japaridze N Lordkipanidze T Rzayev F MacFabe D Zhvania M . Behavioural and brain ultrastructural changes following the systemic administration of propionic acid in adolescent male rats. Further development of a rodent model of autism. Int J Dev Neurosci. (2020) 80:139–56. doi: 10.1002/jdn.10011 31997401

[139] Guo X Huang C Xu J Xu H Liu L Zhao H . Gut microbiota is a potential biomarker in inflammatory bowel disease. Front Nutr. (2021) 8:818902. doi: 10.3389/fnut.2021.818902 35127797 PMC8814525

[140] Sharma B Agriantonis G Twelker K Ebelle D Kiernan S Siddiqui M . Gut microbiota serves as a crucial independent biomarker in inflammatory bowel disease (ibd). Int J Mol Sci. (2025) 26:2503. doi: 10.3390/ijms26062503 40141145 PMC11942158

[141] Cardoneanu A Mihai C Rezus E Burlui A Popa I Cijevschi Prelipcean C . Gut microbiota changes in inflammatory bowel diseases and ankylosing spondilytis. J Gastrointestin Liver Dis. (2021) 30:46–54. doi: 10.15403/jgld-2823 33548121

[142] Ning L Zhou YL Sun H Zhang Y Shen C Wang Z . Microbiome and metabolome features in inflammatory bowel disease via multi-omics integration analyses across cohorts. Nat Commun. (2023) 14:7135. doi: 10.1038/s41467-023-42788-0 37932270 PMC10628233

[143] Lawal SA Voisin A Olof H Bording-Jorgensen M Armstrong H . Diversity of the microbiota communities found in the various regions of the intestinal tract in healthy individuals and inflammatory bowel diseases. Front Immunol. (2023) 14:1242242. doi: 10.3389/fimmu.2023.1242242 38022505 PMC10654633

[144] Bajaj A Markandey M Kedia S Ahuja V . Gut bacteriome in inflammatory bowel disease: An update on recent advances. Indian J Gastroenterol. (2024) 43:103–11. doi: 10.1007/s12664-024-01541-1 38374283

[145] Hu Y Chen Z Xu C Kan S Chen D . Disturbances of the gut microbiota and microbiota-derived metabolites in inflammatory bowel disease. Nutrients. (2022) 14:5140. doi: 10.3390/nu14235140 36501169 PMC9735443

[146] Park N Lee B Jeon HJ Yeon Kim J Park S Kim SE . Inulin-butyrate nanogel for modulation of gut microbiome, intestinal barrier, and regulatory t-cells in colitis. Small. (2026) 22:e13252. doi: 10.1002/smll.202513252 41854082 PMC13173310

[147] Grellier N Suzuki MT Brot L Rodrigues AMS Humbert L Escoubeyrou K . Impact of ibd-associated dysbiosis on bacterial quorum sensing mediated by acyl-homoserine lactone in human gut microbiota. Int J Mol Sci. (2022) 23:15404. doi: 10.3390/ijms232315404 36499731 PMC9738069

[148] Ekstedt N Jamioł-Milc D Pieczyńska J . Importance of gut microbiota in patients with inflammatory bowel disease. Nutrients. (2024) 16:2092. doi: 10.3390/nu16132092 38999840 PMC11242987

[149] Chen L Ruan G Cheng Y Yi A Chen D Wei Y . The role of th17 cells in inflammatory bowel disease and the research progress. Front Immunol. (2022) 13:1055914. doi: 10.3389/fimmu.2022.1055914 36700221 PMC9870314

[150] Guo Z Zhang J Liu X Unsinger J Hotchkiss RS Cao YQ . Low-dose interleukin-2 reverses chronic migraine-related sensitizations through peripheral interleukin-10 and transforming growth factor beta-1 signaling. Neurobiol Pain. (2022) 12:100096. doi: 10.1016/j.ynpai.2022.100096 35733705 PMC9207571

[151] Michaud E Waeckel L Gayet R Goguyer-Deschaumes R Chanut B Jospin F . Alteration of microbiota antibody-mediated immune selection contributes to dysbiosis in inflammatory bowel diseases. EMBO Mol Med. (2022) 14:e15386. doi: 10.15252/emmm.202115386 35785473 PMC9358401

[152] Bjørklund G Pivina L Dadar M Meguid NA Semenova Y Anwar M . Gastrointestinal alterations in autism spectrum disorder: What do we know? Neurosci Biobehav Rev. (2020) 118:111–20. doi: 10.1016/j.neubiorev.2020.06.033 32621940

[153] Lee JG Cho HJ Jeong YM Lee JS . Genetic approaches using zebrafish to study the microbiota-gut-brain axis in neurological disorders. Cells. (2021) 10:566. doi: 10.3390/cells10030566 33807650 PMC8002147

[154] Eshraghi RS Davies C Iyengar R Perez L Mittal R Eshraghi AA . Gut-induced inflammation during development may compromise the blood-brain barrier and predispose to autism spectrum disorder. J Clin Med. (2020) 10:27. doi: 10.3390/jcm10010027 33374296 PMC7794774

[155] Darwesh MK Bakr W Omar TEI El-Kholy MA Azzam NF . Unraveling the relative abundance of psychobiotic bacteria in children with autism spectrum disorder. Sci Rep. (2024) 14:24321. doi: 10.1038/s41598-024-72962-3 39414875 PMC11484847

[156] Huang YY Li CY Li Y Fang H Ke XY . Characteristics and functions of the gut microbiome in monozygotic twins with autism spectrum disorders of varying severity. World J Psychiatry. (2026) 16:111012. doi: 10.5498/wjp.v16.i2.111012 41641220 PMC12865458

[157] Ding X Xu Y Zhang X Zhang L Duan G Song C . Gut microbiota changes in patients with autism spectrum disorders. J Psychiatr Res. (2020) 129:149–59. doi: 10.1016/j.jpsychires.2020.06.032 32912596

[158] Petropoulos A Stavropoulou E Tsigalou C Bezirtzoglou E . Microbiota gut-brain axis and autism spectrum disorder: Mechanisms and therapeutic perspectives. Nutrients. (2025) 17:2984. doi: 10.3390/nu17182984 41010510 PMC12472681

[159] Cupaioli FA Mosca E Magri C Gennarelli M Moscatelli M Raggi ME . Assessment of haptoglobin alleles in autism spectrum disorders. Sci Rep. (2020) 10:7758. doi: 10.1038/s41598-020-64679-w 32385356 PMC7210291

[160] Ahmadani A Kittana M Al-Marzooq F Subramanya SB D'Adamo MC Attlee A . Zinc ion dyshomeostasis in autism spectrum disorder. Nutr Res Rev. (2025) 38:661–81. doi: 10.1017/s095442242500006x 40079191

[161] Takada R Toritsuka M Yamauchi T Ishida R Kayashima Y Nishi Y . Granulocyte macrophage colony-stimulating factor-induced macrophages of individuals with autism spectrum disorder adversely affect neuronal dendrites through the secretion of pro-inflammatory cytokines. Mol Autism. (2024) 15:10. doi: 10.1186/s13229-024-00589-2 38383466 PMC10882766

[162] Ferencova N Visnovcova Z Ondrejka I Hrtanek I Bujnakova I Kovacova V . Peripheral inflammatory markers in autism spectrum disorder and attention deficit/hyperactivity disorder at adolescent age. Int J Mol Sci. (2023) 24:11710. doi: 10.3390/ijms241411710 37511467 PMC10380731

[163] Runge K Tebartz van Elst L Maier S Nickel K Denzel D Matysik M . Cerebrospinal fluid findings of 36 adult patients with autism spectrum disorder. Brain Sci. (2020) 10:355. doi: 10.3390/brainsci10060355 32521749 PMC7349103

[164] Campanale A Mir HD Dumais E Inserra A Flamand N Chakravarty M . Uncovering novel endocannabinoidome-gut microbiome-brain axis-based therapeutic targets in a fragile x syndrome mouse model. Prog Neuro-Psychopharmacol Biol Psychiatry. (2026) 144:111575. doi: 10.1016/j.pnpbp.2025.111575 41386393

[165] Vakilzadeh G Martinez-Cerdeño V . Pathology and astrocytes in autism. Neuropsychiatr Dis Treat. (2023) 19:841–50. doi: 10.2147/ndt.s390053 37077706 PMC10106330

[166] Zahedi E Sadr SS Sanaeierad A Roghani M . Chronic acetyl-l-carnitine treatment alleviates behavioral deficits and neuroinflammation through enhancing microbiota derived-scfa in valproate model of autism. BioMed Pharmacother. (2023) 163:114848. doi: 10.1016/j.biopha.2023.114848 37163781

[167] Arabi TZ Alkattan W Sabbah BN Ouban A . The gatekeepers breached: Claudin dysregulation in psychiatric disorders. Tissue Barriers. (2026) 14:2616111. doi: 10.1080/21688370.2026.2616111 41550027 PMC13540084

[168] Wang Y Li N Yang JJ Zhao DM Chen B Zhang GQ . Probiotics and fructo-oligosaccharide intervention modulate the microbiota-gut brain axis to improve autism spectrum reducing also the hyper-serotonergic state and the dopamine metabolism disorder. Pharmacol Res. (2020) 157:104784. doi: 10.1016/j.phrs.2020.104784 32305492

[169] Zhong JG Lan WT Feng YQ Li YH Shen YY Gong JH . Associations between dysbiosis gut microbiota and changes of neurotransmitters and short-chain fatty acids in valproic acid model rats. Front Physiol. (2023) 14:1077821. doi: 10.3389/fphys.2023.1077821 37035670 PMC10073564

[170] Lagod P Naser S . The role of short-chain fatty acids and altered microbiota composition in autism spectrum disorder: A comprehensive literature review. Int J Mol Sci. (2023) 24:17432. doi: 10.3390/ijms242417432 38139261 PMC10743890

[171] Zhai L Huang C Ning Z Zhang Y Zhuang M Yang W . Ruminococcus gnavus plays a pathogenic role in diarrhea-predominant irritable bowel syndrome by increasing serotonin biosynthesis. Cell Host Microbe. (2023) 31:33–44.e35. doi: 10.1016/j.chom.2022.11.006 36495868

[172] Peralta-Marzal LN Prince N Bajic D Roussin L Naudon L Rabot S . The impact of gut microbiota-derived metabolites in autism spectrum disorders. Int J Mol Sci. (2021) 22:10052. doi: 10.3390/ijms221810052 34576216 PMC8470471

[173] Mou QH Zhang Q Chen L Dai Y Wei H Jia FY . Gender specific influence of serotonin on core symptoms and neurodevelopment of autism spectrum disorders: A multicenter study in china. Child Adolesc Psychiatry Ment Health. (2025) 19:35. doi: 10.1186/s13034-025-00892-7 40158142 PMC11954278

[174] Chakraborti B Verma D Guhathakurta S Jaiswal P Singh AS Sinha S . Gender-specific effect of 5-ht and 5-hiaa on threshold level of behavioral symptoms and sex-bias in prevalence of autism spectrum disorder. Front Neurosci. (2019) 13:1375. doi: 10.3389/fnins.2019.01375 31998058 PMC6961535

[175] Bermudez-Martin P Becker JAJ Caramello N Fernandez SP Costa-Campos R Canaguier J . The microbial metabolite p-cresol induces autistic-like behaviors in mice by remodeling the gut microbiota. Microbiome. (2021) 9:157. doi: 10.1186/s40168-021-01103-z 34238386 PMC8268286

[176] Lalanne S Fougerou-Leurent C Anderson GM Schroder CM Nir T Chokron S . Melatonin: From pharmacokinetics to clinical use in autism spectrum disorder. Int J Mol Sci. (2021) 22:1490. doi: 10.3390/ijms22031490 33540815 PMC7867370

[177] Pellegrini C Antonioli L Calderone V Colucci R Fornai M Blandizzi C . Microbiota-gut-brain axis in health and disease: Is nlrp3 inflammasome at the crossroads of microbiota-gut-brain communications? Prog Neurobiol. (2020) 191:101806. doi: 10.1016/j.pneurobio.2020.101806 32473843

[178] Osama A Anwar AM Ezzeldin S Ahmed EA Mahgoub S Ibrahim O . Integrative multi-omics analysis of autism spectrum disorder reveals unique microbial macromolecules interactions. J Adv Res. (2025) 77:265–79. doi: 10.1016/j.jare.2025.01.036 39870302 PMC12627878

[179] Heidari A Rostam-Abadi Y Rezaei N . The immune system and autism spectrum disorder: Association and therapeutic challenges. Acta Neurobiol Exp (Wars). (2021) 81:249–63. doi: 10.21307/ane-2021-023 34672295

[180] Jamar G Ribeiro D Pisani L . High-fat or high-sugar diets as trigger inflammation in the microbiota-gut-brain axis. Crit Rev Food Sci Nutr. (2021) 61:836–54. doi: 10.1080/10408398.2020.1747046 32267169

[181] Fasipe B Laher I . Obesity and metabolic disease in migrants: A role for the gut microbiome? Front Clin Diabetes Healthc. (2025) 6:1745885. doi: 10.3389/fcdhc.2025.1745885 41613388 PMC12846935

[182] Soldán M Argalášová L Hadvinová L Galileo B Babjaková J . The effect of dietary types on gut microbiota composition and development of non-communicable diseases: A narrative review. Nutrients. (2024) 16:3134. doi: 10.3390/nu16183134 PMC11434870 39339734

[183] Di Rosa C Di Francesco L Spiezia C Khazrai YM . Effects of animal and vegetable proteins on gut microbiota in subjects with overweight or obesity. Nutrients. (2023) 15:2675. doi: 10.3390/nu15122675 37375578 PMC10300930

[184] Shen Y Meng L Li Y Yang J . Bacteroides ovatus has the potential to be a next-generation probiotic strain. Probiotics Antimicrob Proteins. (2026) 18:2305–15. doi: 10.1007/s12602-025-10699-9 40742521

[185] Chen Z Xiao C Zhang J Jian S Li P Lin J . The impact of diet on the colonization of beneficial microbes from an ecological perspective. J Agric Food Chem. (2025) 73:10069–92. doi: 10.1021/acs.jafc.5c02086 40234746

[186] Dang L Li D Mu Q Zhang N Li C Wang M . Youth-derived lactobacillus rhamnosus with prebiotic xylo-oligosaccharide exhibits anti-hyperlipidemic effects as a novel synbiotic. Food Res Int. (2024) 195:114976. doi: 10.1016/j.foodres.2024.114976 39277213

[187] Hassan NE El-Masry SA El Shebini SM Ahmed NH Mehanna NS Abdel Wahed MM . Effect of weight loss program using prebiotics and probiotics on body composition, physique, and metabolic products: Longitudinal intervention study. Sci Rep. (2024) 14:10960. doi: 10.1038/s41598-024-61130-2 38744950 PMC11094057

[188] Tang H Li W Xu Y Zhou Y Hamblin MR Wen X . Gut microbiota modulation: A key determinant of atopic dermatitis susceptibility in children. Front Microbiol. (2025) 16:1549895. doi: 10.3389/fmicb.2025.1549895 40356648 PMC12066585

[189] Danne C Rolhion N Sokol H . Recipient factors in faecal microbiota transplantation: One stool does not fit all. Nat Rev Gastroenterol Hepatol. (2021) 18:503–13. doi: 10.1038/s41575-021-00441-5 33907321

[190] Magier S Morley T Kelly C . Optimizing therapeutic potential of fecal transplant in inflammatory bowel disease. Gastroenterol Clin North Am. (2025) 54:277–93. doi: 10.1016/j.gtc.2024.12.002 40348488

[191] Sadhu S Paul T Yadav N . Therapeutic engineering of the gut microbiome using synthetic biology and metabolic tools: A comprehensive review with e. Coli nissle 1917 as a model case study. Arch Microbiol. (2025) 207:213. doi: 10.1007/s00203-025-04417-w 40767874 PMC12328466

[192] Puurunen MK Vockley J Searle SL Sacharow SJ Phillips JA 3rd Denney WS . Safety and pharmacodynamics of an engineered e. Coli nissle for the treatment of phenylketonuria: A first-in-human phase 1/2a study. Nat Metab. (2021) 3:1125–32. doi: 10.1038/s42255-021-00430-7 34294923

[193] Montgomery T Peipert D Krementsov D . Modulation of multiple sclerosis risk and pathogenesis by the gut microbiota: Complex interactions between host genetics, bacterial metabolism, and diet. Immunol Rev. (2024) 325:131–51. doi: 10.1111/imr.13343 38717158 PMC11338732

[194] Montgomery TL Künstner A Kennedy JJ Fang Q Asarian L Culp-Hill R . Interactions between host genetics and gut microbiota determine susceptibility to cns autoimmunity. Proc Natl Acad Sci USA. (2020) 117:27516–27. doi: 10.1073/pnas.2002817117 33077601 PMC7959502

[195] Wong MK Armstrong E Heirali AA Schneeberger PHH Chen H Cochrane K . Assessment of ecological fidelity of human microbiome-associated mice in observational studies and an interventional trial. mBio. (2025) 16:e0190425. doi: 10.1128/mbio.01904-25 40996271 PMC12607873

[196] Metwaly A Kriaa A Hassani Z Carraturo F Druart C Arnauts K . A consensus statement on establishing causality, therapeutic applications and the use of preclinical models in microbiome research. Nat Rev Gastroenterol Hepatol. (2025) 22:343–56. doi: 10.1038/s41575-025-01041-3 40033063

[197] Jiang Y Jiang S Wang Z Zhu P Zhang J Teng F . Precise probiotic therapy: Advances, bottlenecks, and the road to microbiome-informed nutrition. Gut Microbes. (2026) 18:2623359. doi: 10.1080/19490976.2026.2623359 41617669 PMC12867423

[198] Liu Y Li X Chen Y Yao Q Zhou J Wang X . Fecal microbiota transplantation: Application scenarios, efficacy prediction, and factors impacting donor-recipient interplay. Front Microbiol. (2025) 16:1556827. doi: 10.3389/fmicb.2025.1556827 40201444 PMC11975908

[199] Duan S Wang Y Zhan S Ye Z Luo T Zhou Y . Engineered probiotics: A new era in treating inflammatory bowel disease. J Transl Med. (2025) 23:1223. doi: 10.1186/s12967-025-07271-y 41188868 PMC12584474

[200] Kuhl GC Devoy C Abbas M Da Silva Morais E Tangney M . Single-step chromosomal engineering integrates biocontainment and therapeutic function for regulatory-oriented live biotherapeutic chassis design. Pharmaceutics. (2026) 18:915. doi: 10.3390/pharmaceutics18080915 42654032 PMC13517021

